# The potential public health impact of adolescent 4CMenB vaccination on *Neisseria gonorrhoeae* infection in England: a modelling study

**DOI:** 10.1186/s12889-022-14670-z

**Published:** 2023-01-10

**Authors:** Katharine J. Looker, Ross Booton, Najida Begum, Ekkehard Beck, Jing Shen, Katherine M. E. Turner, Hannah Christensen

**Affiliations:** 1 Research Fellow in Mathematical Epidemiology of Infectious Diseases, Population Health Sciences, Bristol Medical School, University of Bristol, Oakfield House, Oakfield Grove, Bristol, BS8 2BN UK; 2 Senior Research Associate in Mathematical Epidemiology of Infectious Diseases, Bristol Veterinary School, University of Bristol, Bristol, BS40 5DU UK; 3 Freelance Consultant c/o GSK, Wavre, Belgium 1300; 4 Senior Director, Value Evidence and Outcomes, GSK, Wavre, Belgium 1300; 5 Senior Manager, Health Economics, GSK, Wavre, Belgium 1300; 6 Reader in Infectious Disease Epidemiology, Bristol Veterinary School, University of Bristol, Bristol, BS40 5DU UK; 7 Senior Lecturer in Infectious Disease Mathematical Modelling, Population Health Sciences, Bristol Medical School, University of Bristol, Bristol, BS8 2BN UK

**Keywords:** *Neisseria gonorrhoeae*, Gonorrhoea, Vaccination, Public health, Sexually-transmitted infection, England, Humans

## Abstract

**Introduction:**

Diagnoses of gonorrhoea in England rose by 26% between 2018 and 2019. Recent evidence that a vaccine against meningococcal B disease currently offered to infants in the UK (4CMenB) could additionally protect (with 31% efficacy) against gonorrhoea has led to renewed hope for a vaccine. A Phase 2 proof-of-concept trial of 4CMenB vaccination against gonorrhoea in adults is currently underway.

**Objectives:**

To investigate the potential public health impact of adolescent gonorrhoea vaccination in England, considering different implementation strategies.

**Methods:**

We developed a deterministic transmission-dynamic model of gonorrhoea infection among heterosexual 13–64-year-olds stratified by age, sex and sexual activity. We explored the impact of a National Immunisation Programme (NIP) among 14-year-olds for a vaccine with 31% efficacy, 6 years’ duration of protection, and 85% uptake. We also explored how impact might change for varying efficacy (20–50%) and uptake (75–95%), the addition of a catch-up programme, the use of boosters, and varying duration of protection.

**Results:**

An NIP against gonorrhoea could lead to 50,000 (95% credible interval, CrI 31,000-80,000) and 849,000 (95%CrI 476,000-1,568,000) gonorrhoea infections being averted over 10 and 70 years, respectively, in England, for a vaccine with 31% efficacy and 85% uptake. This is equivalent to 25% (95%CrI 17–33%) of heterosexual infections being averted over 70 years. Vaccine impact is predicted to increase over time and be greatest among 13–18-year-olds (39% of infections 95%CrI 31–49% averted) over 70 years. Varying vaccine efficacy and duration of protection had a noticeable effect on impact. Catch-up and booster vaccination increased the short- and long-term impact, respectively.

**Conclusions:**

A partially-effective vaccine against gonorrhoea infection, delivered to 14-year-olds alongside the MenACWY vaccine, could have an important population impact on gonorrhoea. Catch-up and booster vaccination could be considered alongside cohort vaccination to increase impact.

**Supplementary Information:**

The online version contains supplementary material available at 10.1186/s12889-022-14670-z.

## Introduction

Gonorrhoea, caused by *Neisseria gonorrhoeae* (*N. gonorrhoeae*), is the second most prevalent bacterial sexually-transmitted infection globally [1]. The infection represents a significant public health problem particularly because *N. gonorrhoeae* has rapidly developed resistance to every antibiotic used against it to date [2, 3] and sporadic case reports of highly antibiotic resistant *N. gonorrhoeae* strains have led to increasing concerns about future treatment options [4–7]. In the UK [8] and US [9] ceftriaxone monotherapy is currently the recommended first-line regimen for treating uncomplicated gonorrhoea, along with susceptibility testing and careful monitoring for treatment failure. Until recently, dual therapy with ceftriaxone and azithromycin was recommended in these countries in an attempt to combat multi-drug resistant strains, but evidence had suggested that azithromycin resistance was increasing, the azithromycin dose may be insufficient to clear infections, and higher azithromycin doses could accelerate resistance selection [8]. There have been recent reports, however, of gonorrhoea infections that are resistant to ceftriaxone [10].The latest WHO treatment guidelines recommend dual therapy over single therapy, unless local resistance data are able to inform the choice [11]. In England, prior to the SARS-CoV-2 (“COVID-19”) pandemic, the number of cases of gonorrhoea seen in sexual and reproductive health care settings was increasing: in 2019, the number of diagnoses was 70,936, a 26% increase compared to 2018 [12, 13]. Of those with known sexual orientation, 33,853 (~ 50%) diagnoses were in men who have sex with men (MSM), 15,253 in heterosexual men, and 17,826 in heterosexual women. Symptoms, when present, include burning with urination and genital discharge, among others [14]. Possible complications include disseminated infection, epididymitis in men, and pelvic inflammatory disease, chronic pelvic pain, ectopic pregnancy and tubal factor infertility in women [15, 16]. However, infection is frequently asymptomatic, particularly in women, meaning the prevalence of infection is higher than diagnoses alone suggest [14, 15, 17, 18].

An important potential future control option for gonorrhoea is vaccination [19]. Until recently, efforts to develop an effective vaccine were impeded by the highly antigenically-variable surface of *N. gonorrhoeae*, and a lack of suitable animal models [20]. However, *N. gonorrhoeae* shares 80–90% homology of its primary sequences with *N. meningitidis*, a bacterium which can cause meningitis and septicaemia [21]. Thus, a vaccine against *N. meningitidis* could potentially protect against gonorrhoea infection. A retrospective case-control study among individuals aged 15–30 years attending sexual health clinics in New Zealand who were vaccinated with an outer membrane vesicle meningococcal B vaccine MeNZB, found that vaccination provided partial protection (31% efficacy, 95%CI, 24–39%) against gonorrhoea infection [22]. Two studies using 4CMenB vaccine, a protein-based vaccine designed to protect against group B meningococcal disease routinely offered to infants in the UK, have also shown an impact on gonorrhoea. A retrospective case-control study among 16–23-year-olds in New York City and Philadelphia estimated the vaccine efficacy to be 40% (95%CI 23–53%) [23] and a case-control study of the same vaccine in adolescents and young adults in South Australia estimated the two-dose vaccine effectiveness against gonorrhoea at 32.7% (95%CI 8.3–50.6%) [24]. A proof of concept trial of 4CMenB vaccination in adolescents and adults against gonorrhoea is currently underway (ClinicalTrials.gov: NCT04350138 [25]).

The public health impact in England of an adolescent national immunisation programme (NIP) with the *N. meningitidis* vaccine 4CMenB (trade name Bexsero®, GSK) that also provides partial protection against gonorrhoea infection has not been estimated. Also unknown is the optimal implementation strategy: vaccination with 4CMenB is unlikely to provide life-long protection against gonorrhoea [22, 26], so it will be important to factor in the age-related incidence of gonorrhoea in determining the optimal age for vaccination, and consider whether catch-up or booster vaccination would be useful. In 2019, incidence was low in 13–14-year-olds, 1.2 and 10.1 per 100,000 in males and females respectively, but jumped to 199.7 and 334.7 per 100,000 in males and females aged 15–19 years respectively. Incidence peaked in 20–24-year-olds for males (588.4 per 100,000) and females (395.9 per 100,000), declining in older age groups [13].

In England, the existing routine immunisation schedule offers vaccination to teenagers against human papillomavirus (HPV) starting in school year 8 (12–13 years), and against meningococcal disease-causing capsular groups A, C, W and Y (MenACWY) in school years 9 and 10 (13–15 years) [27]. The MenACWY vaccine is the last vaccine given in the childhood NIP, thus this provides an opportunity, logistically, to offer 4CMenB vaccination to help protect individuals against gonorrhoea, before most have commenced sexual activity, but close in age to when individuals are at increased risk of infection. The MenACWY NIP has around 85% uptake [28] plus approximately 40% uptake of time-limited catch-up vaccination of 18–20-year-olds through general practice (GP) [29, 30], with vaccination offered to girls and boys. (The HPV NIP had 88% coverage for the first dose in females before the SARS-CoV-2 pandemic (2018–2019). Coverage is lower in males (71% coverage for the first dose in 2020–2021), but the NIP has only recently been extended to males, with its introduction impacted by the SARS-CoV-2 pandemic [31].)

An explorative modelling study, not specific to any setting, found that adolescent vaccination against *N. gonorrhoeae* could achieve a substantial reduction in gonorrhoea prevalence even with < 50% vaccine efficacy [32]. There has been limited investigation of the impact of a vaccine with both relatively low efficacy and short duration, and of different vaccination implementation strategies. Moreover, for England, the only public health impact of vaccination that has been addressed by modelling to date is for MSM [33]. To address these research gaps, we developed a mathematical model to address key considerations for a 4CMenB adolescent NIP from an English health policy perspective, namely, to explore the population impact, and the optimal implementation strategy, of *N. gonorrhoeae* vaccination with 4CMenB given plausible ranges for efficacy and uptake.

## Methods

### Model

**Fig. 1:**
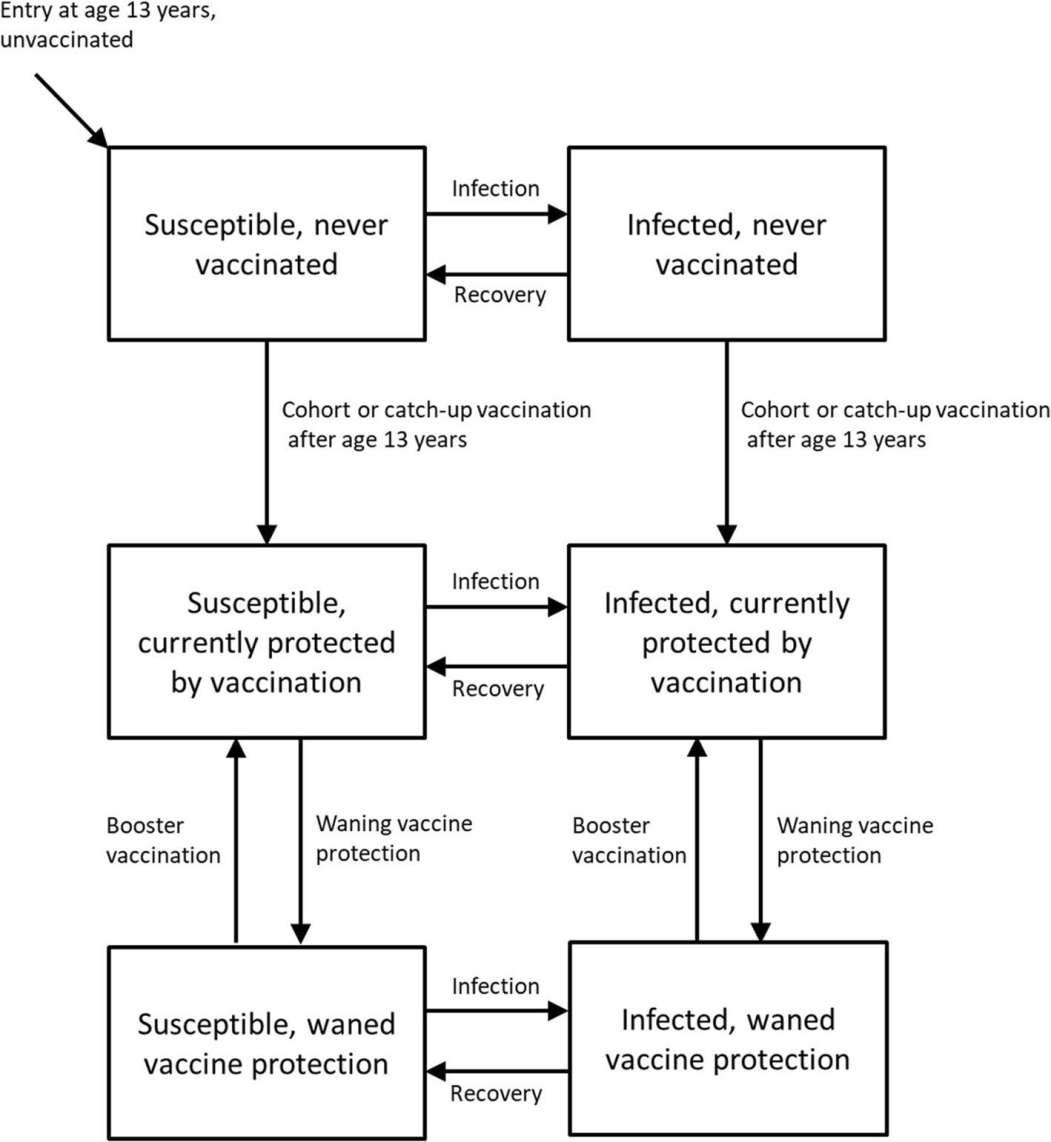
Model flow diagram for gonorrhoea infection and vaccination status

A deterministic, compartmental, transmission-dynamic model of gonorrhoea infection among 13–64-year-olds in England was developed, using the susceptible-infected-susceptible (SIS) model framework, stratified by sex, age, number of sexual partners per year (“sexual activity”), gonorrhoea infection status and vaccination status (Fig. 1, full model details are provided in the Supplementary material). In this simplified representation of the population we stratified the population into two groups, hereon referred to as “women”/“females” and “men”/“males”, and only modelled heterosexual transmission of infection through vaginal intercourse (VI; the most common route of transmission among heterosexual individuals). Since we only modelled heterosexual transmission, we referred to partnerships as occurring with the “opposite sex”. However, terms such as “women”/“females” and “men”/“males”, as relating to gender, represent a range of possible identities, and as such we fully recognize the limitations of the way we have described the model. The model was coded and analysed using R v.3.5.1, and the model ordinary differential equations (ODEs) were solved using deSolve (packages ode and default integrator lsoda). In the model without vaccination, individuals are either susceptible to gonorrhoea infection (i.e., uninfected) or infected with gonorrhoea. Individuals become infected with gonorrhoea at rates determined by their number of sexual partners, the gonorrhoea prevalence among sexual partners, the degree of mixing with each age and sexual activity class, and the probability of gonorrhoea transmission from an infected to a susceptible individual per partnership. Once infected, individuals were assumed to remain infected (and infectious) for a defined period of time before recovering from infection. Even though only heterosexual transmission was modelled, we allowed for importation of infections among men in order to sustain prevalence. In our model, these infections corresponded to infections in MSM who also have sex with women, who acquired infection through sex with other men, and could then transmit the infection to women [34]. The model assumed a constant population over time, and equal numbers of individuals by sex for a given age.

Demographic parameters and the number of imported infections were fixed to point estimates, using, respectively, population data from the Office for National Statistics for mid-2018 [35], and data on gonorrhoea diagnoses among MSM [36] scaled to the percentage of MSM that identify as bisexual as estimated by EMIS 2010 [37] (Table S1, Supplementary material). We defined realistic prior ranges for the rate of recovery from infection [17, 38], and for the transmission probability per partnership by sex in the absence of vaccination [32]. Point estimates and ranges for sexual behaviour parameters were informed by the 3rd National Survey of Sexual Attitudes and Lifestyles (Natsal-3) [39]. The transmission probability per partnership, the rate of recovery, and sexual behaviour parameters were calibrated to data on new gonorrhoea diagnoses among women and men in sexual and reproductive healthcare settings in 2018 [36], which were taken to equal gonorrhoea incidence in heterosexual women and men (“calibration data”) after first adjusting for the number of diagnoses in MSM [36], and adjusting for the fact that not all infections are diagnosed [14, 15, 17, 18, 40] (Table S2, Supplementary material). For some of the sexual behaviour parameters the ranges were adjusted (widened manually) to obtain a more plausible range for the model fit (Table S1, Supplementary material).

The model calibration steps taken were as follows: (1) parameters were sampled from their defined ranges 100,000 times using a Latin Hypercube Sampling approach; (2) the model was simulated from the year 1990 to 2018 (burn-in period) using each of these unique 100,000 parameter sets in turn; (3) 100 parameter sets were selected that generated equilibrium gonorrhoea incidence that best agreed with the calibration data. We calibrated preferentially to infection estimates for women, as there was more uncertainty in the data for men due to the adjustment for diagnoses in MSM. These 100 model fits were used to estimate the posterior for each parameter and these distributions were then used for all model analyses to produce measures of potential vaccine impact (absolute and relative decrease in gonorrhoea incidence compared to baseline) and uncertainty ranges (95% credible intervals, 95%CrI; 2.5th to 97.5th percentiles). Imported infections were not included in the measures of impact. The resultant modelled prevalence was validated against existing prevalence data from Natsal-3 [41].

### Gonorrhoea vaccination

In the model including vaccination, vaccination status was stratified as follows: never vaccinated, currently protected by vaccination, and waned vaccine protection. Cohort vaccination was modelled by moving a percentage (according to vaccine uptake) of individuals out of the never vaccinated compartment into the currently protected by vaccination compartment, at the relevant age, from the year 2018. Vaccine efficacy while in this compartment was modelled as the average reduction in the transmission probability per partnership to vaccinated individuals. Primary vaccine failure (failure of an individual to develop vaccine-induced protection, i.e., “take”) was not explicitly modelled although the vaccine efficacy is an average that will include this (see Supplementary material for full details). After an assumed average duration of vaccine protection (6 years [22, 26], assuming an exponential decline in protection), individuals move out of the currently protected by vaccination model compartment into the waned vaccine protection model compartment. In this compartment, individuals were assumed to have the same probability of gonorrhoea acquisition as those never vaccinated. Individuals remain in the waned vaccine protection for the remainder of their time in the model unless they received a booster vaccination, which places them back into the currently protected by vaccination compartment.

The default (main) scenario was selected to be cohort adolescent vaccination at age 14 years (since the age range for MenACWY vaccination is 13–15-year-olds) with 85% uptake [28] and 31% vaccine efficacy [22]. Both girls and boys were assumed to be vaccinated in equal proportions and independent of sexual activity. The indication for 4CMenB in teenagers against meningococcal group B is a 2-dose schedule with an interval of 1 month; in this model vaccination protection was modelled from the first dose. We also modelled scenarios for varying uptake and vaccine efficacy [22, 23], catch-up vaccination and booster vaccination (Table 1).

**Table 1:** Vaccination scenarios modelled

Scenario	Cohort vaccination	Cohort vaccination characteristics	One-off catch-up	Catch-up vaccination characteristics	Booster	Booster vaccination characteristics
***Main scenario***						
A	14-year-olds	31% VE; 85% VU				
***Catch-up or booster***						
B	14-year-olds	31% VE; 85% VU	15–18 years	31% VE; 40% VU		
C	14-year-olds	31% VE; 85% VU			19–24 years	31% VE; 40% VU
***Varying vaccine uptake***						
D	14-year-olds	31% VE; 75% VU				
E	14-year-olds	31% VE; 95% VU				
***Varying vaccine efficacy***						
F	14-year-olds	20% VE; 85% VU				
G	14-year-olds	50% VE; 85% VU				
***Restricted catch-up***						
H	14-year-olds	31% VE; 85% VU	15–16 years	31% VE; 85% VU		
I	14-year-olds	31% VE; 85% VU	17–18 years	31% VE; 85% VU		

*VE* vaccine efficacy, *VU* vaccine uptake

### Sensitivity analysis

We conducted sensitivity analyses to explore combinations of differing uptake and efficacy for cohort adolescent vaccination on its own, with catch up, and with booster (Scenarios S1–24, Supplementary material). We also explored sensitivity analyses for varying duration of protection, due to the uncertainty here (3 or 10 years’ protection, Scenarios S25 and S26), an additional scenario for 40% efficacy corresponding to the mean vaccine efficacy as reported in the New York City and Philadelphia study (Scenario S27), higher baseline gonorrhoea incidence (a 26% increase in incidence data used to recalibrate the model, corresponding to the observed increase between 2018 and 2019 [12, 13] and resulting in an increase in overall incidence of ~ 22%; Scenario S28), and fewer imported infections (model recalibrated to a 75% decrease, corresponding to the possible 75% decrease in incidence in MSM with 4CMenB suggested by modelling [33], because an adolescent NIP might be accompanied by a vaccination programme for sexually higher-risk populations such as MSM; Scenario S29).

## Results

### Model fits

We visually compared the baseline gonorrhoea incidence by age group and sex for the 100 selected best fitting model runs with the calibration data to assess model fit. The model was preferentially fitted to the data for women and the trend by age group in the modelled gonorrhoea incidence for women closely represented that of the fitting incidence (Fig. S1, Supplementary material). For men modelled incidence similarly was in close agreement for age groups up to 24 years, however in the 25–64 year old group the modelled incidence was less than suggested by data. The median values for model parameters against informing data are shown in Fig. S2 (Supplementary material). Modelled prevalence by age replicated the patterns observed in prevalence data, i.e., highest prevalence in those aged 19–24 years and higher prevalence in women compared to men (Fig. S3, Supplementary material).

### Impact of vaccination

**Fig. 2:**
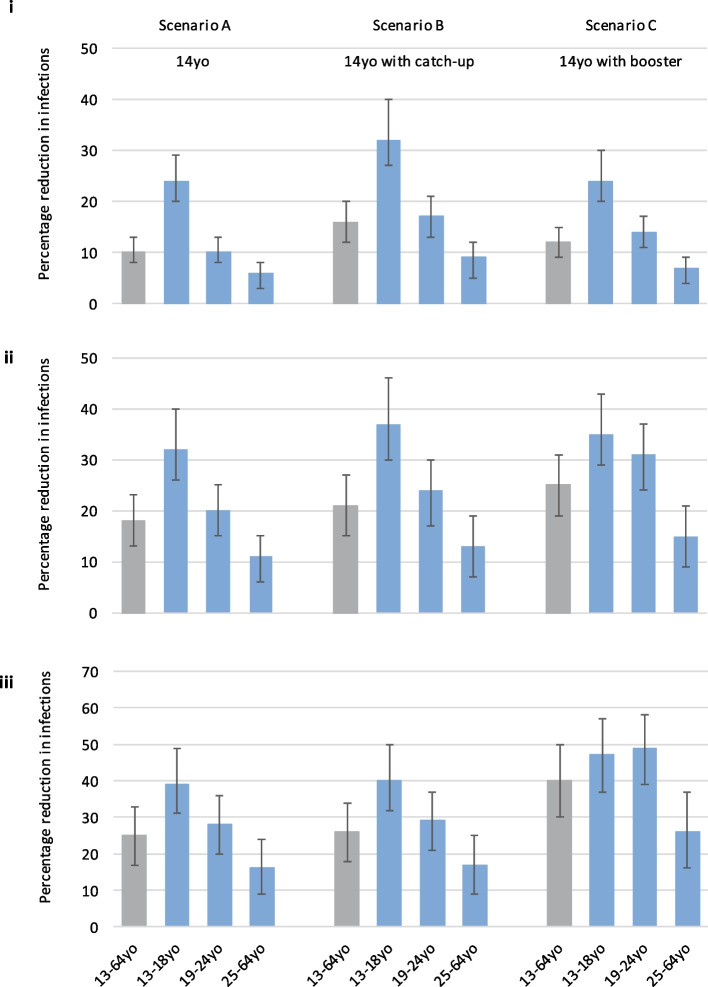
Percentage reduction in incident (new) gonorrhoea infections over 10 (i), 20 (ii) and 70 (iii) years

An adolescent NIP against gonorrhoea with 31% efficacy and 85% uptake among 14-year-olds (Scenario A: main scenario) could lead to 50,000 (95% credible interval, CrI 31,000-80,000), 174,000 (95%CrI 102,000-308,000) and 849,000 (95%CrI 476,000-1,568,000) heterosexual incident (i.e., new) gonorrhoea infections being averted over 10, 20, and 70 years, respectively, among those aged 13–64 years (Supplementary Table S3). This was equivalent to 10% (95%CrI 8–13%), 18% (95%CrI 13–23%) and 25% (95%CrI 17–33%) of heterosexual incident gonorrhoea infections being averted (Fig. 2). Vaccine impact was largest for 13–18-year-olds compared to older individuals in the shorter term, reflecting the direct protection afforded to adolescents by vaccination, with impact increasing over time across all age groups as incidence and prevalence declined (Scenario A, Supplementary Table S3, Fig. 2). For example, over 10 years, 24% (95%CrI 20–29%) of incident gonorrhoea infections were predicted to be averted for 13–18-year-olds compared to 6% (95%CrI 3–8%) for 25–64-year-olds, but over 70 years, 39% (95%CrI 31–49%) and 16% (95%CrI 9–24%) of infections among 13–18 and 25–64-year-olds, respectively, were predicted to be averted.

Figure shows percentage reduction in gonorrhoea infections over time since introduction of a national vaccination programme, by vaccination scenario, for 13–64-year-olds (grey bars, median and 95% credible intervals) and by age group (blue bars, median and 95% credible intervals): vaccination of adolescents (Scenario A); vaccination of adolescents and one-off catch-up (Scenario B); vaccination of adolescents with a booster (Scenario C).

### Catch-up and booster vaccination

**Fig. 3:**
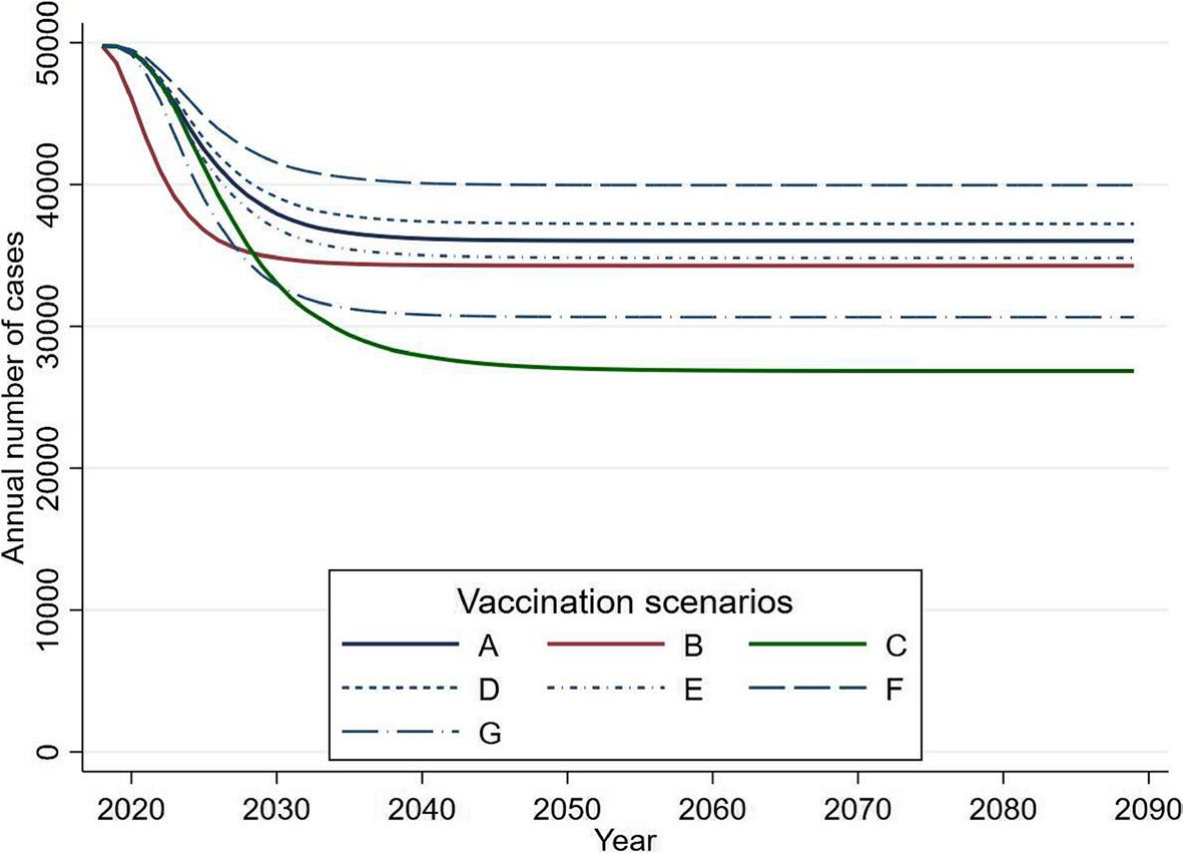
Predicted annual number of gonorrhoea infections (cases) in 13–64-year-olds in England

With the addition of catch-up vaccination for 15–18-year-olds at 40% uptake for 1 year (Scenario B), the short-term gains would be greater than without catch-up (Figs. 2 and 3): over 10 years, 77,000 (95%CrI 47,000–128,000) gonorrhoea infections could be averted among 13–64-year-olds (27,000 or 54% more than without catch-up), which is equivalent to 16% (95%CrI 12–20%) of incident gonorrhoea infections (Fig. 2, Supplementary Table S3). In contrast, booster vaccination for 19–24-year-olds at 40% uptake (Scenario C) could lead to greater long-term gains than without booster vaccination: 1,370,000 (95%CrI 794,000-2,367,000) or 40% (95%CrI 30–50%) of gonorrhoea infections averted over 70 years among 13–64-year-olds (521,000 or ~ 61% more than without booster vaccination). With restricted catch-up vaccination (two out of four yearly cohorts; Scenarios H and I), the increase in the short-term impact (over 10 years) of the vaccine is similar to that for more extensive catch-up (15 and 17% of infections averted respectively, compared to 16% for Scenario B) because the catch-up uptake is assumed to be higher in the restricted catch-up scenarios (85% versus 40% for Scenario B) (Supplementary Table S3).

Figure shows predicted annual number of gonorrhoea infections under different scenarios (Scenarios A-G) for adolescent gonorrhoea vaccination (median values of 100 model runs).

### Varying vaccine uptake and efficacy

Predicted vaccine impact was only marginally less than the baseline scenario for a lower (75%) vaccine uptake (Scenario D): 9% (95%CrI 7–12%) of gonorrhoea infections are predicted to be averted across all ages over 10 years rising to 23% (95%CrI 15–30%) over 70 years, and only slightly greater for higher (95%) uptake (Scenario E) with respective impacts 11% (95%CrI 9–14%) and 27% (95%CrI 19–36%) over 10 and 70 years (Supplementary Table S3, Fig. 3). Varying vaccine efficacy from 20 to 50% had a somewhat larger effect on predicted impact. For 20% vaccine efficacy (Scenario F) 7% (95%CrI 5–9%) and 17% (95%CrI 12–24%) of gonorrhoea infections are predicted to be averted over 10 and 70 years respectively. This increased to 15% (95%CrI 12–19%) and 35% (95%CrI 25–44%) of gonorrhoea infections averted over 10 and 70 years for 50% efficacy (Scenario G). With a vaccine efficacy of 50%, a third (34%, 95%CrI 29–41%) of infections among 13–18-year-olds would be averted over 10 years, rising to over half (53%, 95%CrI 44–63%) over 70 years.

### Sensitivity analysis

The vaccine impact was reduced if efficacy and uptake were simultaneously lower, and increased if efficacy and uptake were simultaneously higher, versus scenarios changing neither or only one of these parameters (Supplementary Table S4, Scenarios S1-S24 versus Supplementary Table S3, Scenarios D-I). A shorter duration of vaccine protection resulted in a reduction in vaccine impact: for example, with a vaccine with an average duration of protection of 3 years, 13% (95%CrI 9–18%) of all incident gonorrhoea infections would be averted over 70 years (Supplementary Table S4, Scenario S25). If the vaccine offered a greater duration of protection (average of 10 years) compared to the baseline scenario, vaccine impact would be greater (Scenario S26, 34% of infections averted CrI 24–39% over 70 years). However, the use of boosters can mitigate short duration of protection. With a vaccine offering 6 years’ average protection but with the use of boosters (Supplementary Table S3, Scenario C), long-term impact is either greater than or similar to (depending on age group) a vaccine with an average duration of protection of 10 years and no boosting (Supplementary Table S4, Scenario S26).

If the vaccine efficacy is 40% rather than 31%, then 171,000 additional infections may be prevented over 70 years (Supplementary Table S4, Scenario S27). With higher incidence (Supplementary Table S4, Scenario S28), the vaccine impact was similar in terms of percentage reduction in gonorrhoea infections compared to the main scenario. With fewer imported infections the vaccine impact was greater (almost twice as many infections averted after 70 years, Supplementary Table S4, Scenario S29, compared to Scenario A), because heterosexual transmission accounted for a greater proportion of infections (due to fitted parameters increasing the transmission probability per partnership per year and decreasing the rate of recovery from infection in males in this scenario).

## Discussion

### Principal findings

Cohort vaccination of 14-year-olds in England with 4CMenB against gonorrhoea assuming 31% efficacy and 85% uptake could lead to a substantial reduction in new gonorrhoea infections, particularly among adolescents. For example, over 10 years, 50,000 (95%CrI 31,000-80,000) or 10% (95%CrI 8–13%) of heterosexual incident gonorrhoea infections could be averted, rising to 25% (95%CrI 17–33%) or 849,000 (95%CrI 476,000-1,568,000) over 70 years. In the short-term (10 years), around 54% more infections could be prevented with the addition of catch-up vaccination. Booster vaccination, meanwhile, could lead to around 61% more infections being prevented in the long-term (70 years), and could help mitigate a short duration of vaccine protection. For a vaccine with higher efficacy (50%), a third of infections among younger ages (13–18-year-olds) could be averted over ten years, increasing to over half of infections over 70 years.

### Strengths and limitations of this study

Our study is the first to our knowledge to explore the impact of an NIP for 4CMenB with partial efficacy on gonorrhoea in the heterosexual English population. The transmission dynamic model incorporates considerable complexity with heterogeneity (and model compartments) by age group, sex and sexual behaviour, to describe the transmission of gonococcal infection. The parameters used are strongly informed by large studies of sexual behaviour and infection rates specific to England. We explored a range of vaccine efficacies that were based directly on study data, and considered a number of possible implementation strategies relevant for policy makers and commissioners. Since our study was conducted, a matched cohort study of the impact of 4CMenB on gonorrhoea infection among teenagers and young adults in Southern California has reported an estimated vaccine efficacy of 46% (95%CI 34–86%), which is in line with that reported by other studies, if slightly higher [42].

There is uncertainty in the underlying data informing our model parameters, particularly around natural history (e.g., rate of recovery, probability of infection, percentage symptomatic), patterns of sexual mixing, and sexual behaviour (in particular among 13–15-year-olds). We incorporated parameter uncertainty in our estimates of impact by sampling from plausible ranges, and used prevalence data to validate our model, but did need to widen the sampling ranges in some age groups to generate a workable model. We compared the effect of key vaccination parameters on model predictions in our analyses. Reassuringly, the predicted impact in terms of percentage reduction in incidence was not sensitive to the base rate of infections though vaccine impact would be greater if the number of imported infections were fewer. The duration of 4CMenB vaccine protection against *N. gonorrhoeae* is unknown. Our base case assumption of an average 6 years’ protection (with waning in an exponential fashion) is based on the observed decline in anti-OMV antibodies over time [26, 43, 44]. This may be conservative given immunity may involve more than just serum bactericidal antibodies. To explore the impact of this parameter we also considered vaccine impact with longer and shorter protection levels.

As with any modelling study, populations are simulated and there are necessarily simplifying assumptions, such as assuming equal population by age between females and males, and applying the same sexual behaviour parameters (but not transmission probabilities) to both sexes. However, this also enables multiple vaccine scenarios and implementation strategies to be compared easily. We did not specifically consider MSM in our model, although we did stratify the model by sexual activity and include importation of infections which approximates bridging from MSM.

The benefits of vaccination may be higher than we have estimated because we only consider transmission through vaginal intercourse (although diagnosis data used for fitting do not specify anatomical location) and have not included possible co-infections, such as with HIV, that could increase transmission rates. In this model we only considered gonorrhoea infection irrespective of accompanying acute symptoms and possible complications - the natural history of disease, and in particular, the risk of longer-term outcomes, is more uncertain than for infection. Reducing associated complications of gonorrhoea infection and reductions in antimicrobial resistance would add further value to an NIP against gonorrhoea.

### Strengths and limitations compared to other studies

A number of other, previous transmission-dynamic models have considered the role of vaccination against gonococcal infection, though in contrast to our work, these have focused on either vaccination in MSM [33, 45] or have been in a heterosexual population, but not focused on a particular geographical setting [32]. Whittles et al. [33] developed a stochastic transmission-dynamic model in MSM in England, considering both antibiotic resistant and sensitive strains and evaluating a large number of hypothetical vaccine profiles. Based on data available at the time, they considered three scenarios using parameters designed to align with properties of the 4CMenB vaccine (2 to 4 year duration of vaccine protection) and found a 7% (0–23%) reduction in incidence in MSM after 10 years with vaccination before sexual debut, assuming no emergence of antibiotic resistance. This is similar to the incidence reduction we find in our heterosexual model where we implement vaccination at age 14 years. Greater vaccine impact was seen when vaccination was implemented at attendance at sexual health clinics (75%, 40–98%) or at gonorrhoea diagnosis (41%, 18–65% assuming 100% vaccine uptake). The authors noted that the before sexual debut strategy in MSM was ineffective, and the vaccination on attendance strategy was insufficient to meet the WHO target of reducing gonorrhoea incidence by 90% during 2018–2030 [46] in 75% of the simulations. Hui et al. [45] also modelled potential vaccine impact of *N. gonorrhoeae* vaccines in an MSM population. The model considered infection across multiple anatomical sites and is tailored to an Australian setting. They estimated considerably larger reductions in infection, with a 62% prevalence reduction within 2 years, but assumed the vaccine has 50% efficacy against infection with 80% of MSM attending STI clinics annually (high compared to a UK context) and 30% of these choosing to be vaccinated. The results of these models highlight the considerable reductions that can be achieved when targeting a higher-risk group, however the impact critically depends on accessing this population to offer vaccination. Craig et al. [32] used an individual based model to estimate the impact of vaccination against gonorrhoea in a general heterosexual population, including complex sexual mixing patterns (regular, short-term, and concurrent partnerships) though not tailored to a specific country. They considered a number of hypothetical vaccine profiles in terms of efficacy and duration of protection with vaccination implemented before sexual debut at 13 years of age. Similarly to our study, the authors found a substantial effect on gonococcal prevalence could occur even with a vaccine that was partially efficacious. They estimated a 40% reduction in prevalence after 20 years with a vaccine of 20% efficacy and 20 years' duration of protection.

### Implications for policy makers (meaning of the study)

A partially-effective vaccine against gonorrhoea infection could have an important population impact on gonorrhoea, and could therefore add substantial value to the existing benefit of the vaccine against invasive meningococcal disease and in combating the spread of antimicrobial resistance. The global health sector strategy [46] called for a 90% reduction in the incidence of *N. gonorrhoeae* by 2030, however the latest WHO global progress report highlighted that increased efforts are needed to achieve this [47]. A sizeable impact on gonorrhoea incidence, particularly among adolescents, is estimated in our models with only 31% vaccine efficacy and a relatively short average duration of vaccine protection. In the UK this programme could be implemented in schools alongside the existing MenACWY vaccine and 3-in-1 Td/IPV booster against tetanus, diphtheria and polio offered to 13–15-year-olds [10]. The 4CMenB vaccine would offer some added protection against non-B capsular groups, as well as direct protection against meningococcal group B, but is unlikely to replace the MenACWY product given the high vaccine-induced protection with the conjugate MenACWY vaccine, particularly against carriage, which is important in teenagers.

Catch-up and booster vaccination could be considered in order to increase the short- and long-term impact of an NIP designed to protect against gonorrhoea. Targeting older age groups for cohort vaccination may offer increased protection at the ages of highest incidence for gonorrhoea, but at the cost of missing vaccination before sexual debut for some individuals and likely reduction in vaccine uptake in older ages, which has been observed in other programmes. Targeting of higher-risk groups, such as MSM, should also be considered, to offer benefits against disease to this community, but also because MSM who also have sex with women can import infections into the heterosexual population; our model suggests vaccination of a heterosexual population is considerably more effective when imported infections are lowered (which could be achieved through vaccinating MSM).

In this study we only considered the benefit of vaccination in terms of gonococcal incidence reduction, however, there would be additional benefits of an adolescent 4CMenB programme in terms of meningococcal disease reduction. Antibiotic resistance is a serious and urgent threat and high levels of resistance have developed against a succession of antibiotic treatments for gonorrhoea. There is emerging resistance to our current last line treatment option for this infection, thus prevention through vaccination offers a vital tool to combat this threat.

### Unanswered questions/future research

Our model suggests that the introduction of adolescent vaccination with 4CMenB would provide an important reduction in gonococcal incidence. To evaluate the full benefit of the vaccine, an extended gonorrhoea model which simultaneously considers the impact of the vaccine on gonorrhoea symptoms, including long-term complications of gonorrhoea infection, and on meningococcal infection would be valuable. Allowing for different sexual behaviour between women and men, whilst still balancing the number of partnerships overall, and further including higher-risk groups such as men who have sex with men, would also be useful. Incorporating the implications for combating antibiotic resistance and exploring the optimum age of vaccination if not aligned with an existing NIP would be beneficial as well. Following the public health impact estimates, a health economic evaluation would be needed to enable the Joint Committee on Vaccination and Immunisation (JCVI) to make decisions regarding recommendations for the vaccine.

## Supplementary Information

**Additional file 1.**

## Data Availability

Results from 100 generated parameters sets for model scenarios are available from KJL ORCID ID: 0000–0002–3375-0807. Model equations, parameter ranges used, and scenarios are presented in the Supplementary material.

## Abbreviations

*N. gonorrhoeae*: *Neisseria gonorrhoeae*
NIP: National Immunisation Programme
MenACWY: Meningococcal conjugate vaccine against capsular groups A, C, W and Y
MSM: Men who have sex with men
