# Supplementary material for: The potential public health impact of adolescent 4CMenB vaccination on Neisseria gonorrhoeae infection in England: a modelling study
Source: BMC Public Health. 2023 Jan 10;23:1. doi: 10.1186/s12889-022-14670-z (PMC9829524; doi:10.1186/s12889-022-14670-z)
Supplement: Supplementary file 1 — Additional file 1. [file 12889_2022_14670_MOESM1_ESM.pdf]

Supplementary material for: The potential public health impact of adolescent 4CMenB vaccination on *Neisseria gonorrhoeae* infection in England: a modelling study

Dr Katharine J Looker, Dr Ross Booton, Dr Najida Begum, Dr Ekkehard Beck, Dr Jing Shen, Dr Katherine M E Turner and Dr Hannah Christensen

Further methods

Model demography and infection

The model was stratified by sex (k=1: females; k=2: males), age (a=1: 13 years; a=2: 14 years; a=3: 15-16 years; a=4: 17-18 years; a=5: 19-24 years; a=6: 25-64 years), sexual activity (defined as 0 [j=0], 1 [j=1], 2-3, [j=2] and 4+ [j=3] opposite-sex sexual partners per year), gonorrhoea infection status (X: susceptible; Y: infected) and vaccination status (v=1: never vaccinated; v=2: currently protected by vaccination; v=3: waned vaccine protection). Individuals enter the model at age 13 years at rate μ_a and exit at age 65 years at a rate γ set to maintain a constant population. For computational reasons the model was defined with a model population per age class corresponding to the number of years spent in that age class; model populations were applied to population numbers to calculate numbers of individuals as needed as an output. It was assumed that all individuals entering the model are susceptible to gonorrhoea infection, i.e., no individuals experience gonorrhoea infection before age 13 years. Recovered individuals move back into the susceptible model compartment: it was assumed that previous infection affords no immunity against reinfection. Treatment was not explicitly modelled: the duration of infection was assumed to be an average over all infections for each sex, whether treated or untreated, symptomatic or asymptomatic.

Sexual behaviour

Individuals enter one of the four sexual activity classes on model entry, and can move between sexual activity classes as they age. The percentage of individuals in each of the four sexual activity classes by age, $\rho_{a,j}$, and the corresponding mean number of opposite-sex sexual partners per year for j=2 and j=3 (j=0 and j=1 simply have 0 partners and 1 partner, respectively), $c_{a,j}$, were informed by data for England from the 3rd National Survey of Sexual Attitudes and Lifestyles (Natsal-3). We used data from 11,357 individuals aged 16-64 years surveyed between 2010-2012 to directly inform $\rho_{a,j}$ and $c_{a,j}$ for a=4-6 (i.e., 17-64 year olds). We applied data for 16 year olds to the 15-16 year old age class (a=3). Model values for $\rho_{a,j}$ were kept the same between 13 and 14 year olds, while model values for $c_{a,j}$ were allowed to vary. We used data on age at first sex for 19-24 year olds to inform $\rho_{a,j}$ for j=0 for 13 and 14 year olds. The distribution of sexually-active 13 and 14 year olds in j=1, j=2 and j=3 was then informed by data on the relative percentage of 15-16 year olds in each of these activity classes. Sexual behaviour parameters were assumed to be the same for women and men in order to balance the total number of partnerships.

Since 13 and 14 year olds were assumed to have the same values of $\rho_{a,j}$, there was no movement between sexual activity classes as individuals aged from 13 to 14 years. Data from Natsal 1-3(1) show a decrease in the percentage of individuals with no sexual partners with age across all ages, an increase in the percentage of individuals with one sexual partner with age across all ages, an increase in the percentage of individuals in j=2 and j=3 up to age 18 years, no clear pattern for the change in the

percentage of individuals in j=2 and j=3 for 19-24 year olds, and a decrease in the percentage of individuals in j=2 and j=3 for 25-64 year olds. Given these patterns, sampled parameter sets for the percentage of individuals within each sexual activity class by age were either accepted or rejected using the following set of rules:

1. Ageing from a=2 to a=3: Reject if any of the following occur:
   - $\rho_{3,0} > \rho_{2,0}$
   - $\rho_{3,1} < \rho_{2,1}$
   - $\rho_{3,2} < \rho_{2,2}$
   - $\rho_{3,3} < \rho_{2,3}$
2. Ageing from a=3 to a=4: Reject if any of the following occur:
   - $\rho_{4,0} > \rho_{3,0}$
   - $\rho_{4,1} < \rho_{3,1}$
   - $\rho_{4,2} < \rho_{3,2}$
   - $\rho_{4,3} < \rho_{3,3}$
3. Ageing from a=4 to a=5: Reject if any of the following occur:
   - $\rho_{5,0} > \rho_{4,0}$
   - $\rho_{5,1} < \rho_{4,1}$
4. Ageing from a=5 to a=6: Reject if any of the following occur:
   - $\rho_{6,0} > \rho_{5,0}$
   - $\rho_{6,1} < \rho_{5,1}$
   - $\rho_{6,2} > \rho_{5,2}$
   - $\rho_{6,3} > \rho_{5,3}$

We also constrained $\rho_{a,0}$ so that the percentages summed to 100% for each age class as the percentages when summed cannot be greater than 100%. Movement between sexual activity classes with age was then defined as follows. Individuals stayed within their activity class where possible. Where there was net loss with ageing for only one sexual activity class (here, because of the above, only possible for j=0), the net loss was simulated simply by distributing lost individuals to the other classes. Where there was net gain with ageing for only one sexual activity class (here, because of the above, only possible for j=1), the net gain was simulated simply by taking individuals from the other classes. Where there was net gain or loss for j=2 and the opposite for j=3, the net gain was simulated by taking individuals preferentially from j=2 or j=3 as appropriate, and the remaining gains fulfilled by taking the individuals from j=0.

The equations for ageing were therefore as follows (shown for susceptible individuals, X; an equivalent set of equations were used for infected individuals, Y):

Equation 1: $ageing\_X^{v}_{k,1,j} = -\alpha_1 X^{v}_{k,1,j}$

Equation 2: $ageing\_X^{v}_{k,2,j} = \alpha_1 X^{v}_{k,1,j} - \alpha_2 X^{v}_{k,2,j}$

Equation 3a: $ageing\_X^{v}_{k,3,0} = \alpha_2 X^{v}_{k,2,0} + \left(\alpha_2 * \left(\rho_{3,0} - \rho_{2,0}\right)/\rho_{2,0} * X^{v}_{k,2,0}\right) - \alpha_3 X^{v}_{k,3,0}$

Equation 3b: $ageing\_X^{v}_{k,3,1-3} = \alpha_2 X^{v}_{k,2,1-3} + \left(\alpha_2 * \left(\rho_{3,1-3} - \rho_{2,1-3}\right)/\rho_{2,0} * X^{v}_{k,2,0}\right) - \alpha_3 X^{v}_{k,3,j}$

Equation 4a: $ageing\_X^{v}_{k,4,0} = \alpha_3 X^{v}_{k,3,0} + \left(\alpha_3 * \left(\rho_{4,0} - \rho_{3,0}\right)/\rho_{3,0} * X^{v}_{k,3,0}\right) - \alpha_4 X^{v}_{k,4,0}$

Equation 4b: $ageing\_X^{v}_{k,4,1-3} = \alpha_3 X^{v}_{k,3,1-3} + \left(\alpha_3 * \left(\rho_{4,1-3} - \rho_{3,1-3}\right)/\rho_{3,0} * X^{v}_{k,3,0}\right) - \alpha_4 X^{v}_{k,4,j}$

If $\rho_{5,2} < \rho_{4,2}$ and $\rho_{5,3} > \rho_{4,3}$ (net gain in *j*=3 and net loss in *j*=2; *j*=3 will fill preferentially from *j*=2) then:

Equation 5a: $ageing\_X^{v}_{k,5,0} = \alpha_4 X^{v}_{k,4,0} + (\alpha_4 * (\rho_{5,0} - \rho_{4,0})/\rho_{4,0} * X^{v}_{k,4,0}) - \alpha_5 X^{v}_{k,5,0}$

Equation 5b: $ageing\_X^{v}_{k,5,1} = \alpha_4 X^{v}_{k,4,1} + (\alpha_4 * MIN[(\rho_{4,0} - \rho_{5,0}), (\rho_{5,1} - \rho_{4,1})]/\rho_{4,0} * X^{v}_{k,4,0}) + (\alpha_4 * ([\rho_{5,1} - \rho_{4,1}] - MIN[(\rho_{4,0} - \rho_{5,0}), (\rho_{5,1} - \rho_{4,1})])/\rho_{4,2} * X^{v}_{k,4,2}) - \alpha_5 X^{v}_{k,5,1}$

Equation 5c: $ageing\_X^{v}_{k,5,2} = \alpha_4 X^{v}_{k,4,2} + (\alpha_4 * (\rho_{5,2} - \rho_{4,2})/\rho_{4,2} * X^{v}_{k,4,2}) - \alpha_5 X^{v}_{k,5,2}$

Equation 5d: $ageing\_X^{v}_{k,5,3} = \alpha_4 X^{v}_{k,4,3} + (\alpha_4 * MIN[(\rho_{4,2} - \rho_{5,2}), (\rho_{5,3} - \rho_{4,3})]/\rho_{4,2} * X^{v}_{k,4,2}) + (\alpha_4 * ([\rho_{5,3} - \rho_{4,3}] - MIN[(\rho_{4,2} - \rho_{5,2}), (\rho_{5,3} - \rho_{4,3})])/\rho_{4,0} * Y^{v}_{k,4,0}) - \alpha_5 X^{v}_{k,5,3}$

If $\rho_{5,2} < \rho_{4,2}$ and $\rho_{5,3} < \rho_{4,3}$ (net loss in *j*=2 and *j*=3; both will fill into *j*=1) then:

Equation 5e: $ageing\_X^{v}_{k,5,0;2-3} = \alpha_4 X^{v}_{k,4,j} + (\alpha_4 * (\rho_{5,j} - \rho_{4,j})/\rho_{4,j} * X^{v}_{k,4,j}) - \alpha_5 X^{v}_{k,5,j}$

Equation 5f: $ageing\_X^{v}_{k,5,1} = \alpha_4 X^{v}_{k,4,1} + (\alpha_4 * (\rho_{5,1} - \rho_{4,1})/(1 - \rho_{4,1}) * (X^{v}_{k,4,0} + X^{v}_{k,4,2} + X^{v}_{k,4,3})) - \alpha_5 X^{v}_{k,5,1}$

If $\rho_{5,2} > \rho_{4,2}$ and $\rho_{5,3} > \rho_{4,3}$ (net gain in *j*=2 and *j*=3; both will fill from *j*=0) then:

Equation 5g: $ageing\_X^{v}_{k,5,0} = \alpha_4 X^{v}_{k,4,0} + (\alpha_4 * (\rho_{5,0} - \rho_{4,0})/\rho_{4,0} * X^{v}_{k,4,0}) - \alpha_5 X^{v}_{k,5,0}$

Equation 5h: $ageing\_X^{v}_{k,5,1-3} = \alpha_4 X^{v}_{k,4,1-3} + (\alpha_4 * (\rho_{5,1-3} - \rho_{4,1-3})/\rho_{4,0} * X^{v}_{k,4,0}) - \alpha_5 X^{v}_{k,5,j}$

If $\rho_{5,2} > \rho_{4,2}$ and $\rho_{5,3} < \rho_{4,3}$ (net gain in *j*=2 and net loss in *j*=3; *j*=2 will fill preferentially from *j*=3) then:

Equation 5i: $ageing\_X^{v}_{k,5,0} = \alpha_4 X^{v}_{k,4,0} + (\alpha_4 * (\rho_{5,0} - \rho_{4,0})/\rho_{4,0} * X^{v}_{k,4,0}) - \alpha_5 X^{v}_{k,5,0}$

Equation 5j: $ageing\_X^{v}_{k,5,1} = \alpha_4 X^{v}_{k,4,1} + (\alpha_4 * MIN[(\rho_{4,0} - \rho_{5,0}), (\rho_{5,1} - \rho_{4,1})]/\rho_{4,0} * X^{v}_{k,4,0}) + (\alpha_4 * ([\rho_{5,1} - \rho_{4,1}] - MIN[(\rho_{4,0} - \rho_{5,0}), (\rho_{5,1} - \rho_{4,1})])/\rho_{4,3} * X^{v}_{k,4,3}) - \alpha_5 X^{v}_{k,5,1}$

Equation 5k: $ageing\_X^{v}_{k,5,2} = \alpha_4 X^{v}_{k,4,2} + (\alpha_4 * MIN[(\rho_{4,3} - \rho_{5,3}), (\rho_{5,2} - \rho_{4,2})]/\rho_{4,3} * X^{v}_{k,4,3}) + (\alpha_4 * ([\rho_{5,2} - \rho_{4,2}] - MIN[(\rho_{4,3} - \rho_{5,3}), (\rho_{5,2} - \rho_{4,2})])/\rho_{4,0} * X^{v}_{k,4,0}) - \alpha_5 X^{v}_{k,5,2}$

Equation 5l: $ageing\_X^{v}_{k,5,3} = \alpha_4 X^{v}_{k,4,3} + (\alpha_4 * (\rho_{5,3} - \rho_{4,3})/\rho_{4,3} * X^{v}_{k,4,3}) - \alpha_5 X^{v}_{k,5,3}$

Equation 6a: $ageing\_X^{v}_{k,6,1} = \alpha_5 X^{v}_{k,5,1} + (\alpha_5 * (\rho_{6,1} - \rho_{5,1})/(1 - \rho_{5,1}) * (X^{v}_{k,5,0} + X^{v}_{k,5,2} + X^{v}_{k,5,3})) - \alpha_6 X^{v}_{k,6,1}$

Equation 6b: $ageing\_X^{v}_{k,6,0;2-3} = \alpha_5 X^{v}_{k,5,0;2-3} + (\alpha_5 * (\rho_{6,j} - \rho_{5,j})/\rho_{5,j} * X^{v}_{k,5,j}) - \alpha_6 X^{v}_{k,6,0;j}$

We assumed mostly assortative mixing with respect to age with some mixing with other age classes (2, 3), grouping the age classes 13, 14, 15-16 and 17-18 years together for the purposes for assortative age mixing, and mostly random (proportionate) mixing with respect to sexual activity class with some

assortative mixing within a class(4). The patterns of mixing by age and sexual activity class were defined by mixing matrices that balanced the “supply and demand” of partnerships. Individuals who were not currently sexually active (i.e., in class j=0) did not contribute to sexual mixing by definition. Condom use and its effect on transmission were not explicitly modelled; however, the transmission probability per partnership is an average that will account for this. We also included a proportionate reduction in transmission among 25-64 year olds (calibrated), assuming a reduction in partner change rates in this age group that is not fully captured by differences in the number of partners by age (e.g., one partner per year could be one new partner each year or the same partner over many years).

Imported infections were assumed to be in the (male) j=3 sexual activity class and distributed by vaccination status according to the percentage currently in each vaccination compartment.

Gonorrhoea vaccination

Vaccination was assumed to protect against acquisition of infection according to the vaccine efficacy. Vaccine efficacy can be defined by both degree and take. Vaccine take is the percentage of vaccinated individuals who develop an adequate immune response (i.e., are effectively vaccinated) that can protect them fully or partially against infection. The degree of protection is the proportionate reduction in the transmission probability per partnership to those individuals who respond to the vaccine (i.e., a proportionate reduction in infection acquisition among vaccinated individuals). In trials, it is generally not possible to differentiate between degree and take. Therefore, we did not distinguish between degree and take in the model, but instead, upon vaccination, moved all vaccinated individuals to the currently protected by vaccination model compartment. Vaccination was modelled using a pulse function, which moves all vaccinated individuals at the specified age from the never vaccinated compartment to the currently protected by vaccination compartment at the same time, rather than as an applied rate over the entire year. Within the currently protected by vaccination compartment an average reduction in the transmission probability per partnership was applied to all vaccinated individuals. This average reduction equated to the vaccine efficacy as measured in trials. It was assumed that vaccination affords no protection against duration of infection in breakthrough infections (which are possible in any scenario where vaccine efficacy is less than 100%), nor against infectivity of vaccinated individuals who acquire infection. Individuals can lose vaccine induced protection over time and as a result move into the waned vaccine protection compartment; the rate of movement assumes an exponential decline in protection based on the average duration of protection. In this compartment, individuals were assumed to have the same probability of gonorrhoea acquisition as those never vaccinated. Individuals remain in the waned vaccine protection for the remainder of their time in the model except for booster scenarios.

Booster vaccination was simulated by moving individuals out of the waned vaccine protection compartment into the currently protected by vaccination model compartment. In practice, individuals in the currently protected by vaccination model compartment would also receive the booster (as booster vaccination would be given to any individual previously vaccinated). In the model, such individuals simply remain in the currently protected model compartment. It was assumed that booster vaccination has no effect on the degree or duration of the existing protection of individuals in the currently protected model compartment, i.e., “boosted” individuals already protected by vaccination exit into the waned vaccine protection model compartment at the same rate as individuals who do not receive a booster and the vaccine efficacy does not change. Boosting only begins when those who were the first cohort to receive vaccination reach the age individuals are first eligible to be boosted (19 years; year 2023).

The proportion of individuals susceptible to gonorrhoea at time t is denoted by

$X_{k,a,j}^{v}(t)$

where k denotes sex (k=1: females; k=2: males), a denotes age (a=1: 13 years; a=2: 14 years; a=3: 15-16 years; a=4: 17-18 years; a=5: 19-24 years; a=6: 25-64 years), j denotes sexual activity class in terms of numbers of opposite-sex sexual partners per year (j=0: 0; j=1: 1; j=2: 2-3; j=3: 4+) and v denotes vaccination status (v=1: never vaccinated; v=2: currently protected by vaccination; v=3: waned vaccine protection).

Similarly, the proportion of individuals currently infected with gonorrhoea at time t is denoted by $Y_{k,a,j}^{v}(t)$.

Susceptible, never vaccinated

Equation 7a: $\frac{dX_{k,a,j}^{1}(t)}{dt} = ageing\_X_{k,a,j}^{v} + \mu_a \rho_{a,j} - \lambda_{k,a,j}^{1} X_{k,a,j}^{1}(t) + \delta_k Y_{k,a,j}^{1}(t) - \varphi_a^{1} X_{k,a,j}^{1} - \left(\frac{Y_{k,a,j}^{1}}{(Y_{k,a,j}^{1}+Y_{k,a,j}^{2}+Y_{k,a,j}^{3})}\right) * \frac{I_{k,a,j}}{N_{k,a,j}}$

where $ageing\_X_{k,a,j}^{v}$ is the net change due to ageing, μ_a denotes the rate of model entry, $\rho_{a,j}$ denotes the percentage of individuals in each sexual activity class, $\lambda_{k,a,j}^{v}$ denotes the force of (gonorrhoea) infection, δ_k denotes the rate of recovery from infection, φ_a^{1} denotes the cohort or catch-up vaccination uptake, $I_{k,a,j}$ represents imported infections (among bisexual men into j=3 sexual activity class) and $N_{k,a,j}$ denotes the total number of individuals.

Susceptible, currently protected by vaccination

Equation 7b: $\frac{dX_{k,a,j}^{2}(t)}{dt} = ageing\_X_{k,a,j}^{v} - \lambda_{k,a,j}^{2} X_{k,a,j}^{2}(t) + \delta_k Y_{k,a,j}^{2}(t) - \omega X_{k,a,j}^{2} + \varphi_a^{1} X_{k,a,j}^{1} + \psi_a^{3} X_{k,a,j}^{3} - \left(\frac{Y_{k,a,j}^{2}}{(Y_{k,a,j}^{1}+Y_{k,a,j}^{2}+Y_{k,a,j}^{3})}\right) * \frac{I_{k,a,j}}{N_{k,a,j}}$

where ω is the rate at which vaccine protection wanes and ψ_a^{3} is the booster vaccination uptake.

Susceptible, waned vaccine protection

Equation 7c: $\frac{dX_{k,a,j}^{3}(t)}{dt} = ageing\_X_{k,a,j}^{v} - \lambda_{k,a,j}^{3} X_{k,a,j}^{3}(t) + \delta_k Y_{k,a,j}^{3}(t) + \omega X_{k,a,j}^{2} - \psi_a^{3} X_{k,a,j}^{3} - \left(\frac{Y_{k,a,j}^{3}}{(Y_{k,a,j}^{1}+Y_{k,a,j}^{2}+Y_{k,a,j}^{3})}\right) * \frac{I_{k,a,j}}{N_{k,a,j}}$

Infected, never vaccinated

Equation 8a: $\frac{dY_{k,a,j}^{1}(t)}{dt} = ageing\_Y_{k,a,j}^{v} + \lambda_{k,a,j}^{1} X_{k,a,j}^{1}(t) - \delta_k Y_{k,a,j}^{1}(t) - \varphi_a^{1} Y_{k,a,j}^{1} + \left(\frac{Y_{k,a,j}^{1}}{(Y_{k,a,j}^{1}+Y_{k,a,j}^{2}+Y_{k,a,j}^{3})}\right) * \frac{I_{k,a,j}}{N_{k,a,j}}$

where $ageing\_Y_{k,a,j}^{v}$ is the net change due to ageing.

Infected, currently protected by vaccination

Equation 8b: $\frac{dY_{k,a,j}^{2}(t)}{dt} = ageing\_Y_{k,a,j}^{v} + \lambda_{k,a,j}^{2} X_{k,a,j}^{2}(t) - \delta_k Y_{k,a,j}^{2}(t) - \omega Y_{k,a,j}^{2} + \varphi_a^{1} Y_{k,a,j}^{1} + \psi_a^{3} Y_{k,a,j}^{3} + \left(\frac{Y_{k,a,j}^{2}}{(Y_{k,a,j}^{1}+Y_{k,a,j}^{2}+Y_{k,a,j}^{3})}\right) * \frac{I_{k,a,j}}{N_{k,a,j}}$

Infected, waned vaccine protection

Equation 8c: $\frac{dY^3_{k,a,j}(t)}{dt} = ageing\_Y^v_{k,a,j} + \lambda^3_{k,a,j}X^3_{k,a,j}(t) - \delta_k Y^3_{k,a,j}(t) + \omega Y^2_{k,a,j} - \psi^3_a Y^3_{k,a,j} + \left(\frac{Y^3_{k,a,j}}{(Y^1_{k,a,j}+Y^2_{k,a,j}+Y^3_{k,a,j})}\right) * \frac{I_{k,a,j}}{N_{k,a,j}}$

Force of gonorrhoea infection

The force of infection, $\lambda^v_{k,a,j}$, is defined as follows

Equation 9: $\lambda^v_{k,a,j} = \beta_k(1-\theta^v)c_{a,j}\sum_{l=1}^{3}\sum_{d=1}^{6}\zeta_{a,d,j,l}\left(\frac{Y_{k'd,l}}{N_{k',d,l}}\right)$

where β_k is the transmission probability per partnership per year, θ^v is the average proportionate reduction in the transmission probability per partnership as a consequence of vaccination, $c_{a,j}$ is the annual number of VI sexual partners (assumed to be the same for both women and men), and $\sum_{l=1}^{3}\sum_{d=1}^{6}\zeta_{a,d,j,l}\left(\frac{Y_{k'd,l}}{N_{k',d,l}}\right)$ is the weighted prevalence of infection among partners of opposite sex k' of age class d and in sexual activity class l (l=1, l=2 and l=3 only). Sexual mixing with opposite-sex partners by age and sexual activity class is defined by the mixing matrix $\zeta_{a,d,j,l}$ (which again is the same for women and men). This in turn is the product of the mixing matrix $\zeta_{a,d}$ which defines mixing by age class, and mixing matrix $\zeta_{j,l}$ which defines mixing by sexual activity class.

The mixing matrix $\zeta_{a,d}$ is given by

Equation 10: $\zeta_{a,d} = \left((1-\varepsilon_1)\frac{\sum_{l=1}^{3}c_{d,l}N_{d,l}}{\sum_{l=1}^{3}\sum_{d=1}^{5}c_{d,l}N_{d,l}} + \varepsilon_1\sigma_{ad}\right)$

where ε_1 is the degree of assortative mixing for mixing by age class (2, 3) if 1 represents fully assortative mixing and 0 represents fully random (proportionate) mixing, and σ_{ad} is the identity matrix such that $\sigma_{ad} = 1$ for $a = d$ and $\sigma_{ad} = 0$ when $a \neq d$. Defining the mixing matrix as above means that mixing takes into account differences in population numbers by age.

Similarly, the mixing matrix $\zeta_{j,l}$ is given by

Equation 11: $\zeta_{j,l} = \left((1-\varepsilon_2)\frac{\sum_{d=1}^{6}c_{d,l}N_{d,l}}{\sum_{l=1}^{3}\sum_{d=1}^{6}c_{d,l}N_{d,l}} + \varepsilon_2\sigma_{jl}\right)$

where ε_2 is the degree of assortative mixing for mixing by sexual activity class (2, 4).

For a list of parameter symbols and descriptions please refer to Table S1. For calibration and validation data please refer to Table S2.

The model was coded and analysed using R v.3.5.1, and the model ODEs were solved using deSolve (packages ode and default integrator lsoda). The time step used was one month. Ageing was done at the start of each calendar year. Vaccination (cohort, catch-up or booster) was done halfway through each calendar year. At all other timesteps waning vaccination protection occurred at a rate adjusted to account for the fact that waning did not occur when either ageing or vaccination occurred.

Model analysis

We modelled the following vaccination scenarios:

Cohort adolescent vaccination of 14 year olds:
Vaccine efficacy 20%; vaccine uptake 75% [S1]

Vaccine efficacy 31%; vaccine uptake 75% [D]
Vaccine efficacy 50%; vaccine uptake 75% [S2]
Vaccine efficacy 20%; vaccine uptake 85% [F]
Vaccine efficacy 31%; vaccine uptake 85% [Main Scenario, A]
Vaccine efficacy 50%; vaccine uptake 85% [G]
Vaccine efficacy 20%; vaccine uptake 95% [S3]
Vaccine efficacy 31%; vaccine uptake 95% [E]
Vaccine efficacy 50%; vaccine uptake 95% [S4]

Cohort adolescent vaccination of 14 year olds (vaccine uptake 85%) plus one-off catch-up for 15-18 year olds:
Vaccine efficacy 20%; vaccine uptake for catch-up 30% [S5]
Vaccine efficacy 31%; vaccine uptake for catch-up 30% [S6]
Vaccine efficacy 50%; vaccine uptake for catch-up 30% [S7]
Vaccine efficacy 20%; vaccine uptake for catch-up 40% [S8]
Vaccine efficacy 31%; vaccine uptake for catch-up 40% [B]
Vaccine efficacy 50%; vaccine uptake for catch-up 40% [S9]
Vaccine efficacy 20%; vaccine uptake for catch-up 50% [S10]
Vaccine efficacy 31%; vaccine uptake for catch-up 50% [S11]
Vaccine efficacy 50%; vaccine uptake for catch-up 50% [S12]

Cohort adolescent vaccination of 14 year olds (vaccine uptake 85%) plus booster:
Vaccine efficacy 20%; vaccine uptake for booster 30% [S13]
Vaccine efficacy 31%; vaccine uptake for booster 30% [S14]
Vaccine efficacy 50%; vaccine uptake for booster 30% [S15]
Vaccine efficacy 20%; vaccine uptake for booster 40% [S16]
Vaccine efficacy 31%; vaccine uptake for booster 40% [C]
Vaccine efficacy 50%; vaccine uptake for booster 40% [S17]
Vaccine efficacy 20%; vaccine uptake for booster 50% [S18]
Vaccine efficacy 31%; vaccine uptake for booster 50% [S19]
Vaccine efficacy 50%; vaccine uptake for booster 50% [S20]

Cohort adolescent vaccination of 14 year olds (vaccine uptake 85%) plus one-off catch-up for 15-16 year olds:
Vaccine efficacy 20%; vaccine uptake for catch-up 85% [S21]
Vaccine efficacy 31%; vaccine uptake for catch-up 85% [H]
Vaccine efficacy 50%; vaccine uptake for catch-up 85% [S22]

Cohort adolescent vaccination of 14 year olds (vaccine uptake 85%) plus one-off catch-up for 17-18 year olds:
Vaccine efficacy 20%; vaccine uptake for catch-up 85% [S23]
Vaccine efficacy 31%; vaccine uptake for catch-up 85% [I]
Vaccine efficacy 50%; vaccine uptake for catch-up 85% [S24]

Cohort adolescent vaccination of 14 year olds (vaccine uptake 85%) with varying duration of protection:
Vaccine efficacy 31%; duration of protection 3 years [S25]
Vaccine efficacy 31%; duration of protection 10 years [S26]

Cohort adolescent vaccination of 14 year olds with alternate vaccine efficacy:
Vaccine efficacy 40%; vaccine uptake 85% [S27]

Cohort adolescent vaccination of 14 year olds (vaccine uptake 85%) with higher baseline gonorrhoea incidence:
Vaccine efficacy 31%; +26% incident gonorrhoea infections per year among heterosexual women and men used for fitting (resulting in an increase in overall incidence of ~22%) [S28]

Cohort adolescent vaccination of 14 year olds (vaccine uptake 85%) with lower importation of infections:
Vaccine efficacy 31%; -75% imported infections [S29]

Table S1: Parameters used in the model, default values and ranges used, data sources, and assumptions

Parameter description	Parameter symbol	Values from literature (95%CI) [range]	Point estimate or prior range used for sampling	Point estimate or median estimate in 100 best model fits	Data source(s), assumed distribution for prior range (where relevant)	Notes and assumptions
**Demography**						
Rate of model entry	μ_a	--	--	a=1: 1/104<br>a=2: 0<br>a=3: 0<br>a=4: 0<br>a=5: 0<br>a=6: 0	Values selected to maintain a constant population. Point estimates used in model.	Entry is into the susceptible, never vaccinated class.
Rate of ageing out of an age class	α_a	--	--	a=1: 1<br>a=2: 1<br>a=3: 1/2<br>a=4: 1/2<br>a=5: 1/6<br>a=6: γ=1/40	Values selected to maintain a constant population. Point estimates used in model.	
Number of individuals	$N_{k,a}$	k=1, a=1: 315,480<br>k=1, a=2: 310,434<br>k=1, a=3: 598,851<br>k=1, a=4: 621,221<br>k=1, a=5: 2,084,544<br>k=1, a=6: 14,521,992<br>k=2, a=1: 315,480<br>k=2, a=2: 310,434<br>k=2, a=3: 598,851<br>k=2, a=4: 621,221<br>k=2, a=5: 2,084,544<br>k=2, a=6: 14,521,992	--	k=1, a=1: 315,480<br>k=1, a=2: 310,434<br>k=1, a=3: 598,851<br>k=1, a=4: 621,221<br>k=1, a=5: 2,084,544<br>k=1, a=6: 14,521,992<br>k=2, a=1: 315,480<br>k=2, a=2: 310,434<br>k=2, a=3: 598,851<br>k=2, a=4: 621,221<br>k=2, a=5: 2,084,544<br>k=2, a=6: 14,521,992	Mid-2018 population estimates for England, by single year of age(5). Point estimates used in model.	Assumed to be the same for women and men.
**Sexual behaviour**						
Percentage of individuals in each sexual activity class	$\rho_{a,j}$	a=1, j=0: 91.640%<br>a=2, j=0: 91.640%<br>a=3, j=0: 55.166% (48.28%, 61.73%)<br>a=4, j=0: 33.651% (29.45%, 38.02%)<br>a=5, j=0: 16.558% (14.67%, 18.56%)	a=1, j=0: 100% minus the others in age class<br>a=2, j=0: 100% minus the others in age class<br>a=3, j=0: 100% minus the others in age class<br>a=4, j=0: 100% minus the others in age class	a=1, j=0: 95.59%<br>a=2, j=0: 95.59%<br>a=3, j=0: 73.26%<br>a=4, j=0: 33.651%<br>a=5, j=0: 16.558%<br>a=6, j=0: 14.874%<br>a=1, j=1: 2.46%<br>a=2, j=1: 2.46%<br>a=3, j=1: 15.10%	3rd National Survey of Sexual Attitudes and Lifestyles (Natsal-3): bespoke data analysis, limited to those participants from England, provided by Dr Cath Mercer. Data from 11,357 individuals aged 16-64 years surveyed between 2010-2012 used. Uniform distribution	Assumed to be the same for women and men. Data for 16 year olds applied to those aged 15-16 years in the model. Percentage of 13-14 year olds with 0 partners calculated from survival function

Parameter description	Parameter symbol	Values from literature (95%CI) [range]	Point estimate or prior range used for sampling	Point estimate or median estimate in 100 best model fits	Data source(s), assumed distribution for prior range (where relevant)	Notes and assumptions
		a=6, j=0: 14.874% (14.15%, 15.63%)<br>a=1, j=1: 4.694%<br>a=2, j=1: 4.694%<br>a=3, j=1: 25.173% (19.72%, 31.47%)<br>a=4, j=1: 35.848% (31.53%, 40.22%)<br>a=5, j=1: 52.631% (50.02%, 55.25%)<br>a=6, j=1: 75.584% (74.69%, 76.47%)<br>a=1, j=2: 2.825%<br>a=2, j=2: 2.825%<br>a=3, j=2: 15.149% (11.538%, 19.639%)<br>a=4, j=2: 22.613% (19.704%, 25.813%)<br>a=5, j=2: 19.270% (17.374%, 21.319%)<br>a=6, j=2: 6.955% (6.361%, 7.600%)<br>a=1, j=3: 0.841%<br>a=2, j=3: 0.841%<br>a=3, j=3: 4.512% (2.759%, 7.297%)<br>a=4, j=3: 7.888% (6.214%, 9.965%)<br>a=5, j=3: 11.541% (9.994%, 13.293%)<br>a=6, j=3: 2.587% (2.231%, 2.999%)	a=5, j=0: 100% minus the others in age class<br>a=6, j=0: 100% minus the others in age class<br>a=1, j=1: 0 - 4.694%<br>a=2, j=1: 0 - 4.694%<br>a=3, j=1: 0 - 25.173%<br>a=4, j=1: 35.848%<br>a=5, j=1: 52.631%<br>a=6, j=1: 75.584%<br>a=1, j=2: 0 - 2.825%<br>a=2, j=2: 0 - 2.825%<br>a=3, j=2: 0 - 15.149%<br>a=4, j=2: 22.613%<br>a=5, j=2: 19.270%<br>a=6, j=2: 6.955%<br>a=1, j=3: 0 - 0.841%<br>a=2, j=3: 0 - 0.841%<br>a=3, j=3: 0 - 4.512%<br>a=4, j=3: 7.888%<br>a=5, j=3: 11.541%<br>a=6, j=3: 2.587%	a=4, j=1: 35.848%<br>a=5, j=1: 52.631%<br>a=6, j=1: 75.584%<br>a=1, j=2: 1.52%<br>a=2, j=2: 1.52%<br>a=3, j=2: 9.12%<br>a=4, j=2: 22.613%<br>a=5, j=2: 19.270%<br>a=6, j=2: 6.955%<br>a=1, j=3: 0.41%<br>a=2, j=3: 0.41%<br>a=3, j=3: 3.47%<br>a=4, j=3: 7.888%<br>a=5, j=3: 11.541%<br>a=6, j=3: 2.587%	assumed in model for prior range where not point estimate. The percentage in age class a=1 is taken to be 100% minus the percentages in all other age classes for a given sexual activity class. If, in the rare case that $\rho_{a,j}$ for j=1-3 sum to >100%, this is rejected.	for age at first sex for 19-24 year olds (i.e., percentage not having started sex at age 13 years and at age 14 years (averaged for the two ages), among 19-24 year olds). Percentages of 13-14 year olds in the remaining classes then calculated to be proportionate to those for 15-16 year olds, i.e., percentage in each sexual activity class for 15-16 year olds applied to the estimate of the percentage of 13-14 year olds who have started sex.
Mean number of opposite-sex VI sexual partners per year	$c_{a,j}$	a=1, j=0: 0<br>a=2, j=0: 0<br>a=3, j=0: 0<br>a=4, j=0: 0<br>a=5, j=0: 0	a=1, j=0: 0<br>a=2, j=0: 0<br>a=3, j=0: 0<br>a=4, j=0: 0<br>a=5, j=0: 0	a=1, j=0: 0<br>a=2, j=0: 0<br>a=3, j=0: 0<br>a=4, j=0: 0<br>a=5, j=0: 0	3rd National Survey of Sexual Attitudes and Lifestyles (Natsal-3): bespoke data analysis, limited to those participants from England, provided by Dr Cath	Assumed to be the same for women and men. Data for 16 year olds applied to those aged 13-14 years and

Parameter description	Parameter symbol	Values from literature (95%CI) [range]	Point estimate or prior range used for sampling	Point estimate or median estimate in 100 best model fits	Data source(s), assumed distribution for prior range (where relevant)	Notes and assumptions
		a=6, j=0: 0<br>a=1, j=1: 1<br>a=2, j=1: 1<br>a=3, j=1: 1<br>a=4, j=1: 1<br>a=5, j=1: 1<br>a=6, j=1: 1<br>a=1, j=2: 2.323093 (2.186873, 2.459313)<br>a=2, j=2: 2.323093 (2.186873, 2.459313)<br>a=3, j=2: 2.323093 (2.186873, 2.459313)<br>a=4, j=2: 2.346087 (2.274625, 2.417549)<br>a=5, j=2: 2.305603 (2.255927, 2.355279)<br>a=6, j=2: 2.31655 (2.276936, 2.356165)<br>a=1, j=3: 5.735691 (4.158996, 7.312385)<br>a=2, j=3: 5.735691 (4.158996, 7.312385)<br>a=3, j=3: 5.735691 (4.158996, 7.312385)<br>a=4, j=3: 7.451322 (6.052255, 8.850388)<br>a=5, j=3: 7.375787 (6.744536, 8.007037)<br>a=6, j=3: 7.121504 (6.225144, 8.017863)	a=6, j=0: 0<br>a=1, j=1: 1<br>a=2, j=1: 1<br>a=3, j=1: 1<br>a=4, j=1: 1<br>a=5, j=1: 1<br>a=6, j=1: 1<br>a=1, j=2: 2 - 3<br>a=2, j=2: 2 - 3<br>a=3, j=2: 2 - 3<br>a=4, j=2: 2.346087<br>a=5, j=2: 2.305603<br>a=6, j=2: 2.31655<br>a=1, j=3: 3 - 8<br>a=2, j=3: 3 - 8<br>a=3, j=3: 3 - 8<br>a=4, j=3: 7.451322<br>a=5, j=3: 7.375787<br>a=6, j=3: 7.121504	a=6, j=0: 0<br>a=1, j=1: 1<br>a=2, j=1: 1<br>a=3, j=1: 1<br>a=4, j=1: 1<br>a=5, j=1: 1<br>a=6, j=1: 1<br>a=1, j=2: 2.487<br>a=2, j=2: 2.464<br>a=3, j=2: 2.523<br>a=4, j=2: 2.346087<br>a=5, j=2: 2.305603<br>a=6, j=2: 2.31655<br>a=1, j=3: 5.437<br>a=2, j=3: 5.416<br>a=3, j=3: 6.800<br>a=4, j=3: 7.451322<br>a=5, j=3: 7.375787<br>a=6, j=3: 7.121504	Mercer. Data from 11,357 individuals aged 16-64 years surveyed between 2010-2012 used. Uniform distribution assumed in model for prior range where not point estimate.	those aged 15-16 years in the model. The value of $c_{a,j}$ for j=2 is similar across all ages as it can only take a value between 2 and 3 by definition. j=3 was originally defined as 4+ partners per year, but the lower bound for the range was lowered to be >3 in the fitting process.

Parameter description	Parameter symbol	Values from literature (95%CI) [range]	Point estimate or prior range used for sampling	Point estimate or median estimate in 100 best model fits	Data source(s), assumed distribution for prior range (where relevant)	Notes and assumptions
Degree of assortative mixing	ε_1	ε_1=0.653 [0.4 - 0.95]	ε_1=0.653 [0.4 - 0.95]	ε_1=0.5118	Derived from (1 - mean percentage of partnerships with any disassortative mixing) among women and men, for 16-74 year olds using 10-year age-bands, as reported by the 3rd National Survey of Sexual Attitudes and Lifestyles (Natsal-3) 2010-2012: data from 7,832 partnerships for women and 5,964 partnerships for men used(3). 95%CI based on that used in previous gonorrhoea modelling study in the US(2), which was stated to have been informed by the National Survey of Family Growth. Uniform distribution assumed in model.	
	ε_2	ε_2=0.2 [0.06 - 0.94]	ε_2=0.2 [0.06 - 0.94]	ε_2=0.7843	Sexual mixing pattern of sexual health clinic attendees in Seattle, Washington, USA(4). 95%CI based on that used in previous gonorrhoea modelling study in the US(2), which was stated to have been informed by the National Survey of Family Growth. Uniform distribution assumed in model.	Although we used an available study to inform this parameter, this study is not really applicable to our model population. This parameter is largely unknown.
**Gonorrhoea natural history**						
Transmission probability for the acquiring partner per partnership	β_k	k=1: 0.5 - 0.65(6)<br>k=2: 0.19 - 0.53(6)	k=1: 0.5 - 0.65(6)<br>k=2: 0.19 - 0.53(6)	k=1: 0.5785<br>k=2: 0.3873	Uniform distribution assumed in model.	
Proportionate reduction in transmission for 25-64 year olds	βratio	--	0.5 - 1	0.5950	Uniform distribution assumed in model.	

Parameter description	Parameter symbol	Values from literature (95%CI) [range]	Point estimate or prior range used for sampling	Point estimate or median estimate in 100 best model fits	Data source(s), assumed distribution for prior range (where relevant)	Notes and assumptions
Rate of recovery from infection per year	δ_k	k=1: 1 - 3(2, 7)<br>k=2: 1 - 5(2, 7)	k=1: 1 - 3(2, 7)<br>k=2: 1 - 5(2, 7)	k=1: 1.956<br>k=2: 2.904	Range informed by average duration of infection estimates per year for women and men used by the Centers for Disease Control and Prevention(7), and the duration of asymptomatic and symptomatic gonorrhoea infection used in a previous gonorrhoea modelling study in the US(2). Uniform distribution assumed in model.	
Annual number of imported infections	$I_{k,a,j}$	MSM, 13-14 years: 0<br>MSM, 15-19 years: 967<br>MSM, 20-24 years: 4,369<br>MSM, 25-34 years: 11,333<br>MSM, 35-44 years: 5,836<br>MSM, 45-64 years: 3,824	k=2, a=1, j=3: 0<br>k=2, a=2, j=3: 0<br>k=2, a=3, j=3: 8<br>k=2, a=4, j=3: 45<br>k=2, a=5, j=3: 513<br>k=2, a=6, j=3: 2,225<br>and 0 otherwise	k=2, a=1, j=3: 0<br>k=2, a=2, j=3: 0<br>k=2, a=3, j=3: 8<br>k=2, a=4, j=3: 45<br>k=2, a=5, j=3: 513<br>k=2, a=6, j=3: 2,225<br>and 0 otherwise	UKHSA data tables on number of new diagnoses of gonorrhoea in MSM attending GUM/Level 3 and non-GUM/Level 2 services in England(8) in 2018, scaled to model age categories according to the distribution of all male diagnoses by single year of age, and scaled to the percentage of MSM that identify as bisexual (10.6%) as estimated by EMIS 2010: a self-completion online sexual health needs assessment survey promoted on websites aiming to appeal to MSM: 15,500 MSM surveyed from England, Scotland and Wales(9). Point estimates used in model.	Assumed to be at equilibrium. Infections assumed to be for j=3. Age classes for data did not perfectly align with those in model. Assumed to be distributed among unvaccinated and vaccinated compartments according to the existing relative proportions in each.

95%CI – 95% confidence interval; UKHSA – UK Health Security Agency; VI – vaginal intercourse; GUM – genitourinary medicine; MSM – men who have sex with men. k denotes gender (k=1: females; k=2: males), a denotes age (a=1: 13 years; a=2: 14 years; a=3: 15-16 years; a=4: 17-18 years; a=5: 19-24 years; a=6: 25-64 years), j denotes sexual activity class (defined as 0 [j=0], 1 [j=1], 2-3, [j=2] and 4+ [j=3] opposite-sex sexual partners per year). For a full explanation of symbols see text.

Table S2: Fitting and validation data used in the model, values and ranges used, data sources, and assumptions

Data description	Value used in the model (95%CI)	Data source(s)	Notes and assumptions
**Fitting**			
Number of new gonorrhoea diagnoses per year among heterosexual women and men	k=1, a=1: 32<br>k=1, a=2: 95<br>k=1, a=3: 1,081<br>k=1, a=4: 3,388<br>k=1, a=5: 11,077<br>k=1, a=6: 9,857<br>k=2, a=1: 5<br>k=2, a=2: 11<br>k=2, a=3: 360<br>k=2, a=4: 2,007<br>k=2, a=5: 13,431<br>k=2, a=6: 22,589<br>using the following data on number of new gonorrhoea diagnoses (please note difference in age groups between the model and data below):<br>All women, 13-14 years: 74<br>All women, 15-19 years: 4,080<br>All women, 20-24 years: 4,988<br>All women, 25-34 years: 4,129<br>All women, 35-44 years: 1,074<br>All women, 45-64 years: 551<br>All men, 13-14 years: 6<br>All men, 15-19 years: 2,696<br>All men, 20-24 years: 8,615<br>All men, 25-34 years: 16,591<br>All men, 35-44 years: 7,741<br>All men, 45-64 years: 5,162<br>MSM, 13-14 years: 0<br>MSM, 15-19 years: 967<br>MSM, 20-24 years: 4,369<br>MSM, 25-34 years: 11,333<br>MSM, 35-44 years: 5,836<br>MSM, 45-64 years: 3,824	UKHSA data tables on number of new diagnoses of gonorrhoea in women and men attending GUM/Level 3 and non-GUM/Level 2 services in England in 2018(8). Data by single year of age for 13-24 year olds obtained from UKHSA and differ slightly from reported data. The number of diagnoses in 13 year olds and 14 year olds are estimates. Male diagnoses were adjusted by subtracting the estimated number of diagnoses among MSM. Female diagnoses were assumed to be for heterosexual women.<br>To derive estimates of the total number of infections for calibrating the model reported diagnoses were adjusted using 1/the percentage of infections that are diagnosed. Percentage of infections that are diagnosed were based on diagnosis estimates of 89% of symptomatic infections, 40% of asymptomatic infections among women and 9% of asymptomatic infections among men, assuming 37.5% and 67.5% of infections in women and men, respectively, symptomatic(7, 10-13) giving an adjustment factor of 1.71 for women and 2.66 for heterosexual men, assuming that all infections MSM are diagnosed.	Assumed to be at equilibrium.

Validation			
Gonorrhoea infection prevalence	k=1, a=1: 0.0%<br>k=1, a=2: 0.0%<br>k=1, a=3: 0.0%<br>k=1, a=4: 0.0%<br>k=1, a=5: 0.2% (0.1, 0.7%)<br>k=1, a=6: 0.0%<br>k=2, a=1: 0.0%<br>k=2, a=2: 0.0%<br>k=2, a=3: 0.0%<br>k=2, a=4: 0.0%<br>k=2, a=5: 0.1% (0.0, 0.6%)<br>k=2, a=6: 0.0%<br>based on the following data on gonorrhoea prevalence:<br>Women, 16-17 years: 0.0%<br>Women, 18-19 years: 0.0%<br>Women, 20-24 years: 0.2% (0.1, 0.7%)<br>Women, 25-34 years: 0.0%<br>Women, 34-44 years: 0.0%<br>Men, 16-17 years: 0.0%<br>Men, 18-19 years: 0.0%<br>Men, 20-24 years: 0.1% (0.0, 0.6%)<br>Men, 25-34 years: 0.0%<br>Men, 34-44 years: 0.0%	3rd National Survey of Sexual Attitudes and Lifestyles (Natsal-3): 4,550 sexually-experienced individuals aged 16-44 years in Britain tested between 2010-2012(14).	Assumed to be at equilibrium. Age classes for data did not perfectly align with those in model.

95%CI – 95% confidence interval; UKHSA – UK Health Security Agency; VI – vaginal intercourse; ; GUM – genitourinary medicine; MSM – men who have sex with men. k denotes gender (k=1: females; k=2: males), a denotes age (a=1: 13 years; a=2: 14 years; a=3: 15-16 years; a=4: 17-18 years; a=5: 19-24 years; a=6: 25-64 years). For a full explanation of symbols see text.

Further results

Figure S1: Comparison of model baseline gonorrhoea incidence (annual cases per year) with infection data, for women and men, by age group

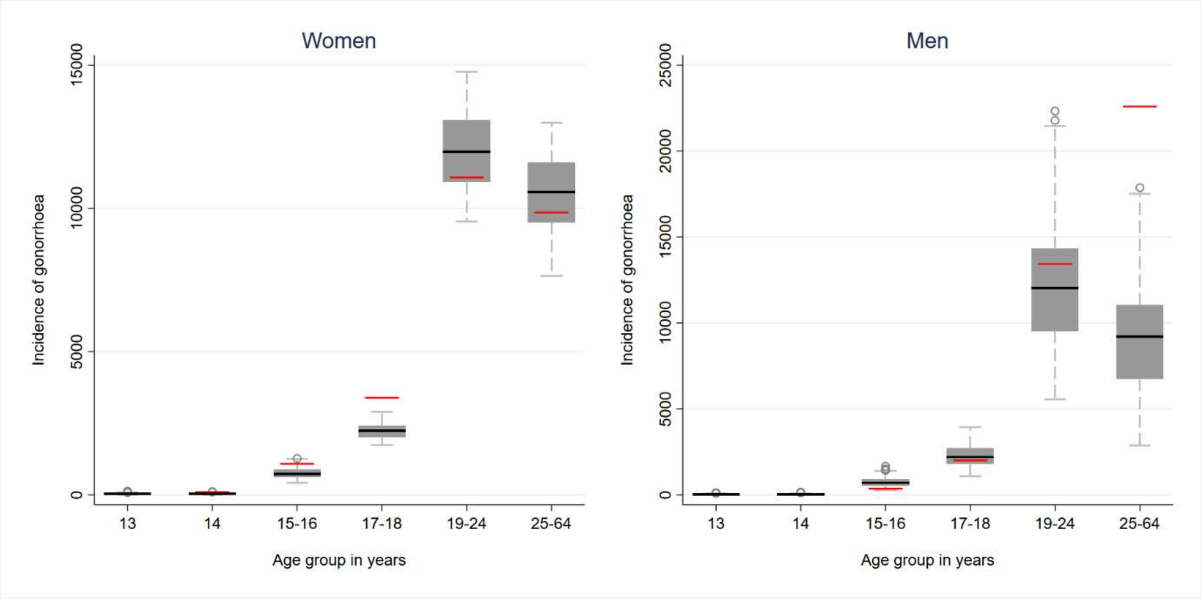

Baseline gonorrhoea incidence (excluding imported infections) for the 100 best model fits is shown by the grey boxplots, with the median value for incidence represented by a black line. The red lines show point estimates of infection incidence for heterosexual women and men from data, adjusted for underreporting.

Figure S2: Comparison of model parameter values with data

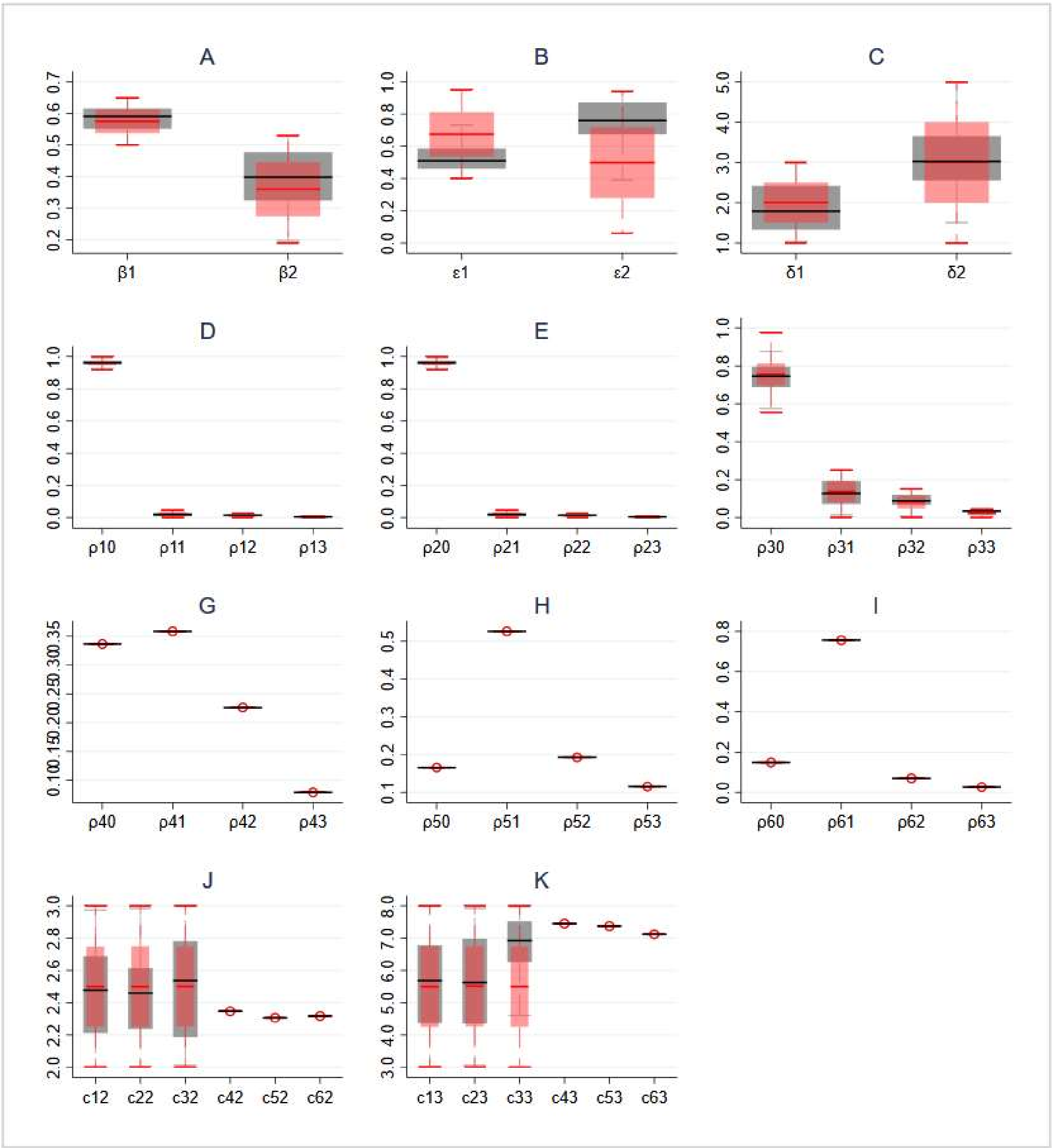

Parameter values for the 100 best model fits are shown by the grey boxplots, with the median value represented by a black line, while the informing data for the parameters is represented by the red boxplots, with the median value represented by a red line. **A** (β_k), transmission, transmission probability per partnership per year in females (k=1) and males (k=2); **B** (ε), epsilon, degree of assortative mixing for mixing by age class (ε_1) and sexual activity class (ε_2); **C** (δ_k), delta, rate of recovery from infection in females (k=1) and males (k=2); **D** ($\rho_{1,j}$), rho1, percentage of individuals in each of four sexual activity classes in age class 1 (13 year olds); **E** ($\rho_{2,j}$), rho2, percentage of individuals in each of four sexual activity classes in age class 2 (14 year olds); **F** ($\rho_{3,j}$), rho3, percentage of individuals in each of four sexual activity classes in age class 3 (15-16 year olds); **G** ($\rho_{4,j}$), rho4, percentage of individuals in each of four sexual activity classes in age class 4 (17-18 year olds); **H** ($\rho_{5,j}$), rho5, percentage of individuals in each of four sexual activity classes in age class 5 (19-24 year olds); **I** ($\rho_{6,j}$), rho6, percentage of individuals in each of four sexual activity classes in age class 6 (25-64 year olds); **J** ($c_{a,2}$), ca2, mean number of opposite-sex sexual partners per year for each of the six age classes for sexual activity class 2 (2-3 opposite-sex sexual partners per year); **K** ($c_{a,3}$), ca3, mean number of opposite-sex sexual partners per year for each of the six age classes for sexual activity class 3 (4+ opposite-sex sexual partners per year).

Figure S3: Model baseline gonorrhoea prevalence, by: **A**, sex; **B**, age group; **C**, age group for women; and **D**, age group for men

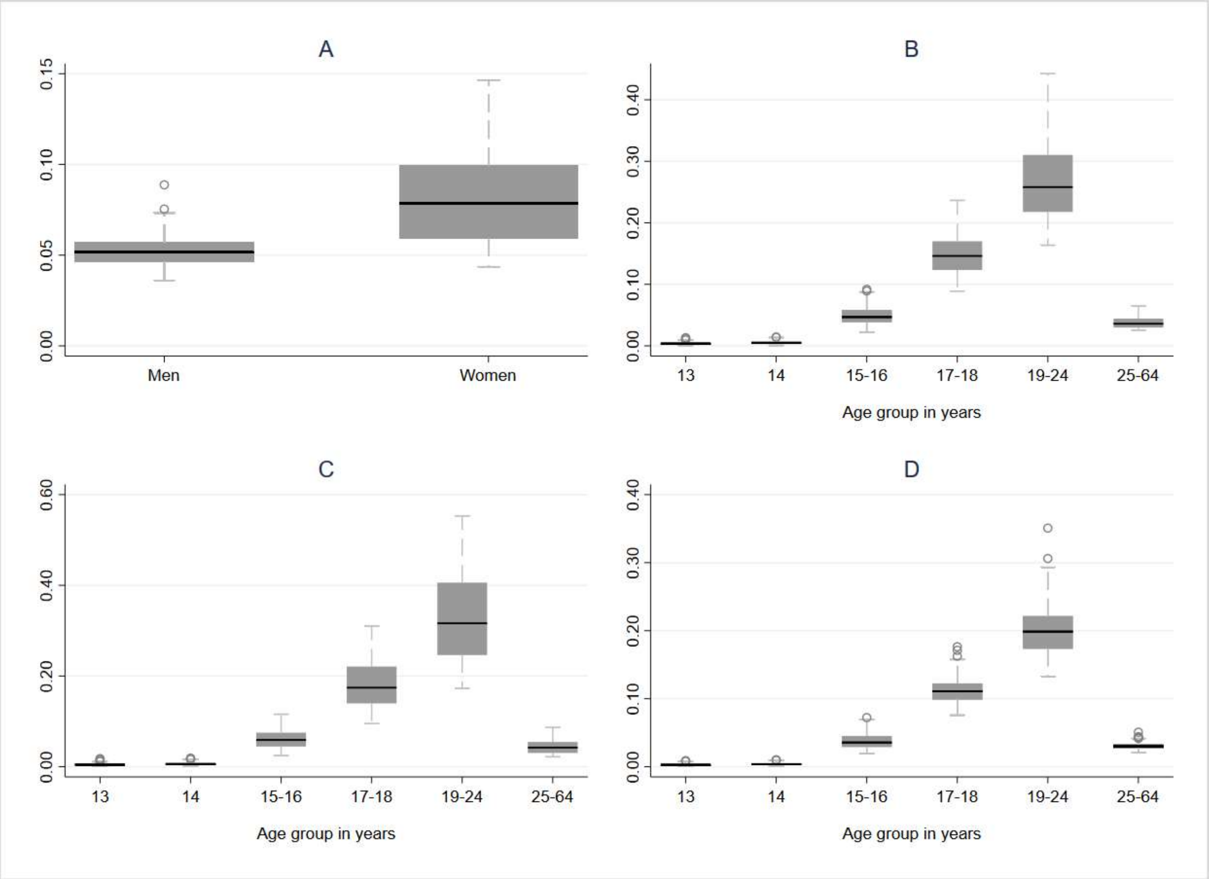

Baseline gonorrhoea prevalence for the 100 best model fits are shown by the grey boxplots, with the median value represented by a black line.

Table S3: Projected model impact of an adolescent gonorrhoea vaccine on the percentage of incident gonorrhoea infections averted in the heterosexual population and number of incident gonorrhoea infections averted over a 10, 20, and 70 year time horizon for different vaccination scenarios, by age

**Vaccination scenario**	***Impact (compared to no vaccine scenario)***	***Time horizon from 2018***											
		10 years Median (95% credible interval)				***20 years Median (95% credible interval)***				***70 years Median (95% credible interval)***			
		Age group (years)											
		All	13-18	19-24	25+	All	13-18	19-24	25+	All	13-18	19-24	25+
**Baseline**	*Total infections, thousands*	495 (352 - 698)	60 (43 - 86)	245 (167 - 348)	194 (135 - 275)	990 (704 – 1,396)	119 (86 - 172)	490 (333 - 696)	388 (269 - 550)	3,467 (2,464 - 4,888)	418 (300 - 601)	1,714 (1,167- 2436)	1,357 (943 – 1,923)
**A** 85% uptake 14yo, 31% vaccine efficacy	*% Infections averted*	10 (8 - 13)	24 (20 -29)	10 (8 - 13)	6 (3 - 8)	18 (13 - 23)	32 (26 - 40)	20 (15 - 25)	11 (6 - 15)	25 (17 - 33)	39 (31 - 49)	28 (20 -36)	16 (9 - 24)
	Infections averted, thousands	50 .(31- 80)	14 (9 - 23)	25 (14- 41)	10 (5- 20)	174 (102 - 308)	38 (24 - 63)	97 (50 -169)	43 (18 - 83)	849 (476 – 1,568)	162 (101 - 274)	476 (243- 890)	222 (90- 450)
**B** 85% uptake 14yo, 40% catch up 15-18yo for 1 year, 31% vaccine efficacy	*% Infections averted*	16 (12 -20)	32 (27- 40)	17 (13- 21)	9 (5- 12)	21 (15- 27)	37 (30- 46)	24 (17- 30)	13 (7- 19)	26 (18 - 34)	40 (32 - 50)	29 (21 -37)	17 (9 - 25)
	Infections averted, thousands	77 (47 -128)	19 (13- 31)	41 (22- 68)	17 (8- 31)	207 (119- 367)	44 (28 - 72)	115 (60- 204)	51 (22- 99)	880 (494 – 1,629)	168 (105 - 283)	494 (252 - 925)	231 (93- 467)
**C** 85% uptake 14yo, 40% booster for previously vaccinated 19-24yo, 31% vaccine efficacy	*% Infections averted*	12 (9- 15)	24 (20- 30)	14 (11- 17)	7 (4 - 9)	25 (19 - 31)	35 (29- 43)	31 (24 - 37)	15 (9 - 21)	40 (30 - 50)	47 (37- 57)	49 (39- 58)	26 (16 -37)
	Infections averted, thousands	60 (38- 96)	15 (9- 24)	33 (19- 53)	12 (6 - 23)	248 (149 - 423)	43 (27 -69)	149 (84 - 249)	60 (27- 112)	1,370 (794 - 2,367)	198 (120 - 323)	836 (469 - 1,414)	369 (159 - 697)
**D** 75% uptake 14yo, 31% vaccine efficacy	*% Infections averted*	9 (7 - 12)	21 (18 -27)	9 (7 - 12)	5 (3-7)	16 (11 - 21)	29 (23- 37)	18 (13- 23)	10 (6 - 14)	23 (15 - 30)	36 (28 - 45)	25 (18 -33)	15 (8- 22)
	Infections averted, thousands	45 (27- 73)	13 (8 - 21)	23 (12 -37)	9 (5 - 18)	157 (92 -280)	35 (22- 58)	87 (45 -154)	39 (16 - 75)	774 (430 - 1,440)	148 (91 -252)	432 (219 - 815)	203 (81 - 411)
**E** 95% uptake 14yo, 31% vaccine efficacy	*% Infections averted*	11 (9 - 14)	26 (22- 32)	11 (9 - 14)	6 (4-8)	19 (14 - 25)	35 (28- 43)	22 (16 -27)	12 (7- 17)	27 (19- 36)	42 (34- 52)	30 (22- 39)	17 (10- 26)
	Infections averted, thousands	55 (34 - 88)	16 (10- 25)	28 (15 -45)	11 (6 - 22)	190 (112- 334)	41 (26 -68)	105 (55 - 184)	47 (20 - 90)	921 (520 - 1,686)	176 (110- 294)	516 (266- 958)	242 (99- 485)
**F** 85% uptake 14yo, 20% vaccine efficacy	*% Infections averted*	7 (5-9)	16 (13- 21)	7 (5 - 9)	4 (2-5)	12 (9- 17)	23 (18- 29)	14 (10- 18)	8 (4-11)	17 (12- 24)	28 (21 - 36)	20 (14- 26)	11 (6 - 17)
	Infections averted, thousands	34 (21- 56)	10 (6- 16)	17 (9- 28)	7 (3- 13)	121 (70- 219)	27 (17- 46)	67 (34- 119)	30 (12 - 58)	608 (329- 1,148)	117 (70 -202)	337 (167- 644)	159 (62 - 324)
**G** 85% uptake 14yo, 50% vaccine efficacy	*% Infections averted*	15 (12- 19)	34 (29- 41)	15 (12- 19)	8 (5- 11)	25 (19 - 32)	45 (38- 53)	29 (22- 35)	16 (9- 22)	35 (25- 44)	53 (44- 63)	39 (29- 48)	23 (13- 32)
	Infections averted, thousands	73 (46- 117)	20 (14- 33)	38 (21- 61)	16 (8- 29)	249 (149 - 427)	54 (35- 85)	138 (75- 236)	60 (27- 117)	1,185 (678- 2.097)	225 (144- 361)	668 (356- 1,193)	315 (132- 613)
**H** 85% uptake 14yo, 85% catch up 15-16yo for 1 year, 31% vaccine efficacy	*% Infections averted*	15 (11 - 19)	32 (26 - 39)	16 (12 - 20)	9 (5 - 12)	21 (15 - 27)	36 (29 - 45)	23 (17 – 30)	13 (7 - 18)	26 (18 - 34)	40 (32 - 50)	29 (21 -37)	17 (9 - 25)
	Infections averted, thousands	74 (45 - 121)	19 (12 - 31)	39 (21 - 65)	16 (8 - 30)	204 (118 - 362)	43 (28 - 72)	113 (59 - 200)	51 (21 - 98)	878 (493 - 1,623)	168 (104 - 283)	493 (252 - 922)	230 (93 - 466)
**I** 85% uptake 14yo, 85% catch up 17-18yo for 1 year, 31% vaccine efficacy	*% Infections averted*	17 (12 -22)	33 (28 -41)	18 (14 -23)	10 (6 - 13)	22 (15 - 28)	37 (30 -46)	24 (18 -31)	13 (8 - 19)	26 (18 - 35)	40 (32 - 51)	29 (21 - 38)	17 (9 - 25)
	Infections averted, thousands	82 (49 -139)	20 (13 -32)	44 (24 -75)	18 (8 - 34)	213 (122 - 378)	44 (28 -73)	118 (61 - 211)	53 (22 - 102)	885 (497 - 1,640)	169 (105 - 284)	498 (254 - 932)	232 (94 - 470)

Table S4: Projected model impact of an adolescent gonorrhoea vaccine on the percentage of incident gonorrhoea infections averted in the heterosexual population and number of incident gonorrhoea infections averted over a 10, 20 and 70 year time horizon for additional vaccination scenarios, by age

Vaccination scenario	*Impact (additional, compared to no vaccine scenario)*	*Time horizon from 2018*											
		10 years Median (95% credible interval)				*20 years Median (95% credible interval)*				*70 years Median (95% credible interval)*			
		Age group (years)											
		All	13-18	19-24	25+	All	13-18	19-24	25+	All	13-18	19-24	25+
**Baseline**	*Total infections, thousands*	495 (352 - 698)	60 (43 - 86)	245 (167 - 348)	194 (135 - 275)	990 (704 – 1,396)	119 (86 - 172)	490 (333 - 696)	388 (269 - 550)	3,467 (2,464 - 4,888)	418 (300 - 601)	1,714 (1,167-2436)	1,357 (943 – 1,923)
**S1** 75% uptake 14yo, 20% vaccine efficacy	*% Infections averted*	6 (5-8)	15 (12-19)	6 (5-8)	3 (2-5)	11 (8-15)	20 (16-26)	12 (9-16)	7 (4-10)	16 (11-22)	25 (19-33)	18 (12-24)	10 (6-16)
	Infections averted, thousands	30 (18-50)	9 (6-14)	15 (8-25)	6 (3-12)	109 (62-197)	24 (15-42)	60 (31-107)	27 (11-52)	549 (294-1043)	106 (63-184)	304 (149-583)	143 (55-193)
**S2** 75% uptake 14yo, 50% vaccine efficacy	*% Infections averted*	13 (10-17)	31 (26-38)	14 (11-17)	8 (5-10)	23 (17-29)	41 (34-50)	26 (20-32)	14 (8-20)	32 (23-41)	49 (40-60)	36 (27-45)	21 (12-30)
	Infections averted, thousands	66 (42-106)	19 (12-30)	34 (19-55)	14 (7-27)	228 (136-394)	49 (32-79)	127 (68-217)	56 (24-107)	1,091 (622-1955)	208 (132-338)	614 (323-1112)	289 (120-569)
**S3** 95% uptake 14yo, 20% vaccine efficacy	*% Infections averted*	8 (6-10)	18 (15-23)	8 (6-10)	4 (3-6)	14 (10-18)	25 (19-32)	15 (11-20)	8 (5-12)	19 (13-26)	30 (23-39)	22 (15-29)	13 (7-19)
	Infections averted, thousands	38 (23-61)	11 (7-18)	19 (10-31)	8 (4-15)	133 (77-239)	30 (18-50)	74 (38-131)	33 (14-64)	663 (362-1247)	127 (77-219)	368 (184-701)	174 (68-353)
**S4** 95% uptake 14yo, 50% vaccine efficacy	*% Infections averted*	16 (13-20)	37 (32-44)	17 (13-20)	9 (6-12)	27 (21-34)	48 (41-57)	31 (24-37)	17 (10-23)	37 (27-47)	57 (48-67)	42 (32-51)	24 (14-34)
	Infections averted, thousands	80 (51-127)	22 (15-35)	41 (23-66)	17 (8-32)	268 (161-457)	58 (38-91)	149 (81-253)	65 (29-125)	1,270 (731-2225)	240 (155-381)	718 (387-1265)	339 (143-654)
**S5** 85% uptake 14yo, 30% catch up 15-18yo for 1 year, 20% vaccine efficacy	*% Infections averted*	10 (7-13)	21 (17-27)	10 (8-13)	6 (3-8)	14 (10-19)	25 (20-33)	16 (11-21)	9 (5-13)	18 (12-25)	29 (22-37)	20 (14-27)	12 (6-18)
	Infections averted, thousands	48 (29-80)	13 (8-21)	25 (14-43)	10 (5-20)	139 (79-252)	30 (19-51)	77 (39-138)	34 (14-67)	635 (338-1181)	120 (72-207)	347 (171-663)	164 (64-333)
**S6** 85% uptake 14yo, 30% catch up 15-18yo for 1 year, 31% vaccine efficacy	*% Infections averted*	14 (11-18)	30 (25-37)	15 (12-19)	8 (5-11)	20 (14-26)	36 (29-44)	23 (17-29)	12 (7-18)	25 (18-34)	40 (32-50)	29 (21-37)	17 (9-25)
	Infections averted, thousands	71 (43-116)	18 (12-29)	37 (20-62)	15 (7-29)	199 (115-354)	43 (27-70)	111 (57-196)	49 (21-95)	873 (490-1614)	167 (104-281)	490 (250-917)	229 (93-463)
**S7** 85% uptake 14yo, 30% catch up 15-18yo for 1 year, 50% vaccine efficacy	*% Infections averted*	21 (16-26)	44 (38-52)	23 (18-28)	12 (7-16)	29 (21-36)	50 (42-59)	32 (25-40)	18 (11-25)	36 (26-46)	55 (45-65)	40 (30-50)	23 (13-33)
	Infections averted, thousands	104 (64-170)	26 (17-41)	55 (31-90)	23 (11-42)	283 (168-489)	60 (39-95)	158 (85-272)	69 (31-134)	1220 (698-2160)	231 (148-370)	687 (366-1229)	324 (135-631)
**S8** 85% uptake 14yo, 40% catch up 15-18yo for 1 year, 20% vaccine efficacy	*% Infections averted*	11 (8-14)	22 (18-28)	12 (9-15)	6 (4-8)	15 (10-20)	26 (20-33)	16 (12-22)	9 (5-13)	18 (12-25)	29 (22-38)	21 (14-27)	12 (6-18)
	Infections averted, thousands	53 (31-88)	14 (9-22)	28 (15-47)	12 (5-22)	144 (82-262)	31 (19-53)	80 (41-144)	36 (15-70)	631 (341-1191)	121 (73-209)	350 (173-670)	165 (64-336)
**S9** 85% uptake 14yo, 40% catch up 15-18yo for 1 year, 50% vaccine efficacy	*% Infections averted*	23 (17-29)	46 (40-55)	25 (19-30)	13 (8-18)	30 (22-37)	51 (43-61)	34 (26-41)	18 (11-26)	36 (26-46)	55 (46-65)	41 (31-50)	23 (14-34)
	Infections averted, thousands	112 (69-195)	28 (19-44)	60 (34-99)	25 (12-46)	293 (173-507)	62 (40-98)	164 (88-282)	72 (32-139)	1230 (703-2178)	233 (149-373)	693 (370-1240)	327 (137-636)

Vaccination scenario	Impact (additional, compared to no vaccine scenario)	*Time horizon from 2018*											
		***10 years** Median (95% credible interval)*				***20 years** Median (95% credible interval)*				***70 years** Median (95% credible interval)*			
		Age group (years)											
		All	13-18	19-24	25+	All	13-18	19-24	25+	All	13-18	19-24	25+
**S10**<br>85% uptake 14yo,<br>50% catch up 15-18yo for 1 year,<br>20% vaccine efficacy	*% Infections averted*	12 (8-15)	24 (19-30)	13 (9-16)	7 (4-9)	15 (11-20)	27 (21-34)	17 (12-22)	9 (5-14)	18 (12-25)	29 (22-38)	21 (14-28)	12 (6-18)
	Infections averted, thousands	57 (34-96)	14 (9-23)	30 (16-52)	13 (6-23)	150 (85-272)	32 (20-54)	83 (42-150)	37 (15-73)	636 (344-1201)	121 (73-210)	353 (175-676)	167 (65-339)
**S11**<br>85% uptake 14yo,<br>50% catch up 15-18yo for 1 year,<br>31% vaccine efficacy	*% Infections averted*	17 (13-22)	34 (28-42)	18 (14-23)	10 (6-13)	22 (16-28)	38 (30-47)	24 (18-31)	13 (8-19)	26 (18-35)	41 (32-51)	29 (21-38)	17 (9-25)
	Infections averted, thousands	83 (50-138)	21 (13-33)	44 (24-74)	19 (9-34)	214 (123-381)	45 (29-74)	119 (62-212)	53 (22-103)	887 (498-1642)	169 (105-285)	499 (255-933)	233 (94-471)
**S12**<br>85% uptake 14yo,<br>50% catch up 15-18yo for 1 year,<br>50% vaccine efficacy	*% Infections averted*	25 (19-31)	49 (42-57)	27 (21-33)	14 (9-19)	31 (23-39)	52 (44-62)	35 (27-43)	19 (11-27)	36 (26-26)	56 (46-66)	41 (31-51)	24 (14-34)
	Infections averted, thousands	121 (74-200)	29 (20-46)	65 (36-107)	27 (13-50)	303 (179-524)	63 (41-100)	170 (92-292)	75 (33-144)	1,240 (709-2195)	235 (150-375)	699 (373-1250)	330 (138-641)
**S13**<br>85% uptake 14yo,<br>30% booster for previously vaccinated 19-24yo,<br>20% vaccine efficacy	*% Infections averted*	8 (6-10)	17 (14-21)	9 (7-11)	4 (3-6)	17 (12-22)	25 (20-32)	21 (16-27)	10 (6-15)	29 (20-37)	34 (26-43)	35 (26-43)	19 (11-27)
	Infections averted, thousands	40 (25-65)	10 (6-17)	22 (12-35)	8 (4-15)	170 (99-297)	30 (18-51)	101 (55-175)	41 (18-78)	974 (551-1770)	144 (84-247)	596 (314-1062)	258 (107-508)
**S14**<br>85% uptake 14yo,<br>30% booster for previously vaccinated 19-24yo,<br>31% vaccine efficacy	*% Infections averted*	12 (9-15)	24 (20-30)	13 (10-16)	6 (4-9)	24 (18-30)	35 (28-43)	29 (23-36)	15 (9-20)	38 (28-48)	46 (36-56)	46 (36-55)	25 (15-35)
	Infections averted, thousands	58 (36-93)	15 (9-24)	32 (18-51)	12 (6-22)	237 (142-406)	42 (26-68)	141 (79-237)	57 (26-108)	1,308 (755-2277)	193 (118-318)	794 (440-1356)	352 (150-668)
**S15**<br>85% uptake 14yo,<br>30% booster for previously vaccinated 19-24yo<br>50% vaccine efficacy	*% Infections averted*	17 (14-21)	35 (30-42)	19 (16-23)	9 (6-13)	33 (26-40)	48 (40-56)	40 (33-46)	20 (13-27)	50 (39-59)	60 (50-69)	60 (50-68)	33 (21-44)
	Infections averted, thousands	86 (54-135)	21 (14-33)	47 (27-74)	18 (9-32)	326 (198-538)	58 (37-90)	194 (113-313)	79 (37-146)	1,718 (1,014-2820)	254 (162-397)	1,029 (607-1670)	462 (210-844)
**S16**<br>85% uptake 14yo,<br>40% booster for previously vaccinated 19-24yo,<br>20% vaccine efficacy	*% Infections averted*	8 (6-11)	17 (14-21)	9 (7-12)	5 (3-6)	18 (13-24)	25 (20-32)	22 (17-28)	11 (6-16)	30 (21-39)	35 (26-44)	37 (28-46)	20 (11-29)
	Infections averted, thousands	42 (25-67)	10 (6-17)	23 (13-37)	8 (4-16)	179 (105-312)	31 (19-51)	108 (59-185)	43 (19-82)	1,030 (584-1860)	148 (87-252)	634 (337-1118)	274 (114-535)
**S17**<br>85% uptake 14yo,<br>40% booster for previously vaccinated 19-24yo,<br>50% vaccine efficacy	*% Infections averted*	18 (14-22)	35 (30-42)	20 (17-24)	10 (6-13)	35 (27-41)	48 (41-57)	41 (35-48)	21 (13-28)	52 (41-61)	61 (51-70)	63 (53-70)	35 (22-46)
	Infections averted, thousands	88 (56-139)	21 (14-33)	49 (29-77)	18 (9-33)	339 (206-556)	58 (38-91)	203 (119-326)	83 (38-151)	1783 (1058-2902)	257 (164-402)	1073 (642-1723)	481 (221-871)
**S18**<br>85% uptake 14yo,<br>50% booster for previously vaccinated 19-24yo,<br>20% vaccine efficacy	*% Infections averted*	9 (7-11)	17 (14-21)	10 (8-12)	5 (3-6)	19 (14-24)	26 (20-33)	23 (18-29)	11 (7-16)	31 (22-40)	36 (27-45)	39 (29-47)	21 (12-30)
	Infections averted, thousands	43 (26-69)	10 (6-17)	24 (13-38)	9 (4-16)	186 (109-324)	31 (19-52)	113 (62-193)	45 (20-85)	1071 (609-1925)	150 (88-257)	662 (355-1160)	286 (119-556)
**S19**<br>85% uptake 14yo,<br>50% booster for previously vaccinated 19-24yo,<br>31% vaccine efficacy	*% Infections averted*	13 (10-16)	25 (20-30)	14 (12-17)	7 (4-9)	26 (20-32)	36 (29-44)	32 (26-39)	16 (10-22)	42 (31-51)	47 (38-57)	51 (41-60)	27 (17-38)
	Infections averted, thousands	62 (39-98)	15 (9-24)	35 (20-55)	13 (6-23)	257 (155-437)	43 (27-70)	155 (88-258)	62 (28-116)	1,416 (824-2433)	201 (122-327)	867 (491-1457)	382 (166-719)
**S20**<br>85% uptake 14yo,	*% Infections averted*	18 (14-22)	35 (30-42)	21 (17-25)	10 (6-13)	36 (28-42)	49 (41-57)	43 (36-50)	22 (14-29)	53 (42-62)	61 (52-70)	65 (55-72)	36 (23-47)

Vaccination scenario	Impact (additional, compared to no vaccine scenario)	Time horizon from 2018											
		10 years Median (95% credible interval)				20 years Median (95% credible interval)				70 years Median (95% credible interval)			
		Age group (years)											
		All	13-18	19-24	25+	All	13-18	19-24	25+	All	13-18	19-24	25+
50% booster for previously vaccinated 19-24yo, 50% vaccine efficacy	*Infections averted, thousands*	91 (58-143)	21 (14-33)	51 (30-80)	19 (9-34)	350 (213-569)	59 (38-92)	210 (124-335)	85 (40-155)	1830 (1091-2961)	260 (166-405)	1105 (667-1761)	495 (229-891)
**S21** 85% uptake 14yo, 85% catch up 15-16yo for 1 year, 20% vaccine efficacy	*% Infections averted*	10 (8 - 13)	22 (18 - 28)	11 (8 - 14)	6 (3 – 8)	15 (10 -19)	26 (20 -33)	16 (12 -21)	9 (5 - 13)	18 (12 - 25)	29 (22 -38)	20 (14 - 27)	12 (6 - 18)
	Infections averted, thousands	51 (31 -84)	13 (8 - 22)	27 (14 -45)	11 (5 -21)	143 (81 - 259)	31 (19 -53)	79 (40 -142)	35 (15 - 69)	629 (340 – 1,189)	120 (73 - 208)	349 (173-668)	165 (64 - 336)
**S22** 85% uptake 14yo, 85% catch up 15-16yo for 1 year, 50% vaccine efficacy	*% Infections averted*	22 (17 -27)	45 (39 - 54)	23 (19 -29)	12 (8 -17)	30 (22 -37)	51 (43 -60)	33 (25 -40)	18 (11 - 25)	36 (26 - 46)	55 (46 -65)	40 (31 - 50)	23 (14 - 33)
	Infections averted, thousands	108 (67 - 175)	27 (18 - 43)	57 (32 - 93)	24 (11 - 44)	289 (171 - 498)	61 (40 -97)	161 (87 - 277)	71 (31 - 137)	1,225 (701 – 2,169)	232 (149 - 372)	691 (368-1,234)	326 (136 - 634)
**S23** 85% uptake 14yo, 85% catch up 17-18yo for 1 year, 20% vaccine efficacy	*% Infections averted*	12 (8 - 15)	23 (19 - 29)	13 (9 - 16)	7 (4 - 9)	15 (10 -20)	26 (21 -34)	17 (12 -22)	9 (5 - 14)	18 (12 - 25)	29 (22 -38)	21 (14 - 28)	12 (6 - 18)
	Infections averted, thousands	56 (33 -95)	14 (9 - 23)	30 (16 -52)	12 (6 -23)	148 (84 - 269)	32 (20 -53)	82 (42 -149)	37 (15 - 72)	634 (343 – 1,198)	121 (73 - 209)	353 (174 - 674)	166 (65 - 338)
**S24** 85% uptake 14yo, 85% catch up 17-18yo for 1 year, 50% vaccine efficacy	*% Infections averted*	25 (19 -31)	48 (41 - 56)	27 (21 -33)	14 (9 -19)	31 (23 -39)	52 (44 -62)	35 (26 -43)	19 (11 - 27)	36 (26 - 46)	55 (46 -66)	41 (31 - 51)	24 (14 - 34)
	Infections averted, thousands	121 (74 - 201)	29 (19 - 44)	65 (36 -109)	27 (13 - 49)	301 (177 - 522)	63 (41 -99)	170 (91 - 292)	74 (33 - 143)	1,238 (707 – 2,194)	234 (150 - 374)	698 (372 - 1,250)	329 (137 - 640)
**S25** 85% uptake 14yo, 31% vaccine efficacy, 3 years' duration	*% Infections averted*	6 (5-8)	16 (13-21)	6 (4-7)	4 (2-5)	10 (7-13)	21 (16-27)	10 (7-13)	6 (3-9)	13 (9-18)	24 (18-32)	14 (9-18)	8 (4-13)
	Infections averted, thousands	30 (18-50)	10 (6-16)	14 (8-23)	6 (3-13)	96 (54-174)	25 (15-43)	49 (24-90)	23 (9-47)	441 (238-863)	102 (61-179)	229 (110-448)	111 (42-237)
**S26** 85% uptake 14yo, 31% vaccine efficacy, 10 years' duration	*% Infections averted*	12 (10-16)	28 (23-35)	13 (10-16)	7 (4-10)	23 (17-27)	38 (31-47)	26 (20-30)	14 (9-19)	34 (24-39)	48 (38-57)	39 (29-42)	23 (13-28)
	Infections averted, thousands	62 (38-98)	17 (11-27)	32 (18-52)	13 (6-24)	226 (134-390)	46 (29-74)	129 (69-219)	56 (25-106)	1149 (654-2037)	201 (125-326)	655 (350-1171)	316 (134-609)
**S27** 85% uptake 14yo, 40% vaccine efficacy	*% Infections averted*	12 (10-16)	29 (24-35)	13 (10-16)	7 (4-10)	22 (16-27)	39 (32-47)	24 (18-30)	13 (8-19)	30 (21-39)	46 (38-57)	34 (25-42)	19 (11-28)
	Infections averted, thousands	61 (38-98)	17 (11-28)	32 (17-51)	13 (6-24)	212 (125-369)	46 (30-75)	118 (62-203)	52 (23-100)	1020 (579-1844)	195 (123-321)	572 (299-1049)	269 (111-534)
**New baseline for +26% baseline incidence**	*Total infections, thousands*	606 (446 - 947)	74 (52 - 119)	300 (210 - 457)	231 (163 - 390)	1212 (892 - 1895)	149 (104 - 238)	599 (420 - 914)	463 (327 - 781)	4242 (3121 - 6638)	521 (364 - 832)	2098 (1470 - 3201)	1620 (1144 - 2737)
**S28** 85% uptake 14yo, 31% vaccine efficacy, +26% baseline incidence	*% Infections averted*	11 (7 - 14)	24 (19 - 31)	11 (8 - 13)	6 (3 - 9)	20 (12 - 24)	33 (26 - 40)	22 (15 - 25)	13 (7 - 17)	28 (17 - 35)	42 (31 - 49)	32 (20 - 38)	18 (10 - 29)
	Infections averted, thousands	67 (32 - 111)	19 (10 - 29)	33 (16 - 54)	14 (6 - 29)	247 (111 - 450)	51 (26 - 84)	133 (61 - 227)	55 (25 - 132)	1215 (535 - 2376)	217 (108 - 387)	663 (299 - 1223)	286 (129 - 730)
**New baseline for -75% imported infections**	*Total infections, thousands*	492 (333-782)	57 (37-96)	241 (160-412)	191 (123-327)	988 (666-1615)	115 (73-196)	484 (321-845)	385 (246-671)	3478 (2335-5932)	407 (256-703)	1,727 (1123-3060)	1,358 (862-2461)
**S29** 85% uptake 14yo, 31% vaccine efficacy, -75% imported infections	*% Infections averted*	13 (9-18)	27 (20-35)	13 (9-17)	9 (5-13)	27 (19-36)	40 (31-50)	29 (21-37)	22 (13-31)	44 (29-58)	56 (41-67)	47 (33-59)	38 (22-53)
	Infections averted, thousands	65 (32-122)	16 (8-28)	32 (16-63)	17 (8-32)	272 (129-518)	49 (24-88)	144 (68-283)	81 (35-161)	1557 (688-3257)	235 (110-468)	812 (368-1812)	497 (198-1146)

References

1. Mercer CH, Tanton C, Prah P, Erens B, Sonnenberg P, Clifton S, et al. Changes in sexual attitudes and lifestyles in Britain through the life course and over time: findings from the National Surveys of Sexual Attitudes and Lifestyles (Natsal). Lancet. 2013;382(9907):1781-94.
2. Ronn MM, Testa C, Tuite AR, Chesson HW, Gift TL, Schumacher C, et al. The potential population-level impact of different gonorrhea screening strategies in Baltimore and San Francisco: an exploratory mathematical modeling analysis. Sex Transm Dis. 2019.
3. Geary RS, Copas AJ, Sonnenberg P, Tanton C, King E, Jones KG, et al. Sexual mixing in opposite-sex partnerships in Britain and its implications for STI risk: findings from the third National Survey of Sexual Attitudes and Lifestyles (Natsal-3). Int J Epidemiol. 2019;48(1):228-42.
4. Garnett GP, Hughes JP, Anderson RM, Stoner BP, Aral SO, Whittington WL, et al. Sexual mixing patterns of patients attending sexually transmitted diseases clinics. Sex Transm Dis. 1996;23(3):248-57.
5. UK Population Estimates 1838 to 2018, Table 11. Population estimates for England, by sex and single year of age, Mid-1971 to Mid-2018 https://www.ons.gov.uk/peoplepopulationandcommunity/populationandmigration/populationestimates/datasets/populationestimatesforukenglandandwalesscotlandandnorthernireland Accessed 22/01/2020.
6. Craig AP, Gray RT, Edwards JL, Apicella MA, Jennings MP, Wilson DP, et al. The potential impact of vaccination on the prevalence of gonorrhea. Vaccine. 2015;33(36):4520-5.
7. Satterwhite CL, Torrone E, Meites E, Dunne EF, Mahajan R, Ocfemia MC, et al. Sexually transmitted infections among US women and men: prevalence and incidence estimates, 2008. Sex Transm Dis. 2013;40(3):187-93.
8. Number of gonorrhoea diagnoses in England by gender, sexual risk & age group, 2014-2018 https://www.gov.uk/government/statistics/sexually-transmitted-infections-stis-annual-data-tables Accessed 24/01/2020.
9. Prah P, Hickson F, Bonell C, McDaid LM, Johnson AM, Wayal S, et al. Men who have sex with men in Great Britain: comparing methods and estimates from probability and convenience sample surveys. Sex Transm Infect. 2016;92(6):455-63.
10. Farley TA, Cohen DA, Elkins W. Asymptomatic sexually transmitted diseases: the case for screening. Prev Med. 2003;36(4):502-9.
11. Institute of Medicine. Vaccines for the 21st Century: A Tool for Decisionmaking. Washington: National Academy Press; 1999.
12. Martin-Sanchez M, Fairley CK, Ong JJ, Maddaford K, Chen MY, Williamson DA, et al. Clinical presentation of asymptomatic and symptomatic women who tested positive for genital gonorrhoea at a sexual health service in Melbourne, Australia. Epidemiol Infect. 2020;148:e240.
13. Owusu-Edusei K, Jr., Chesson HW, Gift TL, Tao G, Mahajan R, Ocfemia MC, et al. The estimated direct medical cost of selected sexually transmitted infections in the United States, 2008. Sex Transm Dis. 2013;40(3):197-201.
14. Sonnenberg P, Clifton S, Beddows S, Field N, Soldan K, Tanton C, et al. Prevalence, risk factors, and uptake of interventions for sexually transmitted infections in Britain: findings from the National Surveys of Sexual Attitudes and Lifestyles (Natsal). Lancet. 2013;382(9907):1795-806.
